# Distinct microRNA Expression Profiles in Mouse Renal Cortical Tissue after ^177^Lu-octreotate Administration

**DOI:** 10.1371/journal.pone.0112645

**Published:** 2014-11-11

**Authors:** Emil Schüler, Toshima Z. Parris, Khalil Helou, Eva Forssell-Aronsson

**Affiliations:** 1 Department of Radiation Physics, Institute of Clinical Sciences, Sahlgrenska Cancer Center, Sahlgrenska Academy at the University of Gothenburg, Sahlgrenska University Hospital, Gothenburg, Sweden; 2 Department of Oncology, Institute of Clinical Sciences, Sahlgrenska Cancer Center, Sahlgrenska Academy at the University of Gothenburg, Sahlgrenska University Hospital, Gothenburg, Sweden; 3 Department of Medical Physics and Biomedical Engineering, Sahlgrenska University Hospital, Gothenburg, Sweden; Oxford Brookes University, United Kingdom

## Abstract

**Aim:**

The aim of this study was to investigate the variation of the miRNA expression levels in normal renal cortical tissue after ^177^Lu-octreotate administration, a radiopharmaceutical used for treatment of neuroendocrine cancers.

**Methods:**

Female BALB/c nude mice were i.v. injected with 1.3, 3.6, 14, 45, or 140 MBq ^177^Lu-octreotate, while control animals received saline. The animals were killed at 24 h after injection and total RNA, including miRNA, was extracted from the renal cortical tissue and hybridized to the Mouse miRNA Oligo chip 4plex to identify differentially regulated miRNAs between exposed and control samples.

**Results:**

In total, 57 specific miRNAs were differentially regulated in the exposed renal cortical tissues with 1, 29, 21, 27, and 31 miRNAs identified per dose-level (0.13, 0.34, 1.3, 4.3, and 13 Gy, respectively). No miRNAs were commonly regulated at all dose levels. miR-194, miR-107, miR-3090, and miR-3077 were commonly regulated at 0.34, 1.3, 4.3, and 13 Gy. Strong effects on cellular mechanisms ranging from immune response to p53 signaling and cancer-related pathways were observed at the highest absorbed dose. Thirty-nine of the 57 differentially regulated miRNAs identified in the present study have previously been associated with response to ionizing radiation, indicating common radiation responsive pathways.

**Conclusion:**

In conclusion, the ^177^Lu-octreotate associated miRNA signatures were generally dose-specific, thereby illustrating transcriptional regulation of radiation responsive miRNAs. Taken together, these results imply the importance of miRNAs in early immunological responses in the kidneys following ^177^Lu-octreotate administration.

## Introduction

Impaired renal function can occur following targeted radionuclide therapy, with risk of nephropathy that may be induced up to 5 years after therapy [1]. Several studies indicate that toxic effects mainly occur in the kidney cortex, and specifically in the proximal tubules [2]–[6]. However, there is limited basic knowledge of how normal kidney tissue responds to ionizing radiation. Although it is widely accepted that response to stressors is mediated by regulation of cell cycle progression and maintenance via gene transcription/translation and epigenetic mechanisms, global cellular responses required for cell survival remain elusive [7]. It is therefore important to have a better understanding of which biological mechanisms play a pivotal role after ionizing radiation exposure in order to provide optimal tumor treatment and minimize normal tissue toxicity.

The kidneys are a late responding organ. However, it is now realized that the “silent interval” between exposure and clinically manifested responses is far from silent. Also in late responding tissue, cytokine cascades are activated and remain active throughout the phase of damage expression. This early release of cytokines gives way for an active biological response mediated by various cell types, including inflammatory, stromal, endothelial and parenchymal cells. As such, data on the early response could very well lead to a better understanding of the biological response following exposure and possibly predict late induced injury [8]–[10]. Recently, the involvement of microRNAs (miRNAs) in response to ionizing radiation was explored [11]. miRNAs are small (18–22 bp), highly conserved non-coding RNAs which bind primarily to the 3′ UTR (untranslated region) of mRNAs and regulate gene expression by promoting mRNA destabilization and degradation as well as repression of translation [7], [12]. Furthermore, miRNA-induced activation of gene expression and thereby enhanced translation has also been proposed [13], [14]. A single miRNA may target multiple mRNAs and several miRNAs may be specific for the same mRNA. The majority of human genes are regulated by different miRNAs, and miRNA play an essential role in fundamental cellular processes, e.g cell metabolism, cell differentiation, apoptosis and cell signaling [15], [16]. Furthermore, miRNAs are involved in cancer differentiation and miRNA expression signatures have been correlated to tumor state and patient clinical outcome [17], [18]. In addition, miRNAs play an important role in the DNA damage response after physiological and pathological stressors, for instance in the response to reactive oxygen species and ionizing radiation [11], [19]–[22].

Previous studies have shown that radionuclide administration has a divergent effect on transcriptional regulation in different normal tissues [23], [24]. Already at very low absorbed doses, organ- and dose-specific responses are seen after ^131^I administration [23]. This has also been shown after ^211^At administration with more pronounced transcriptional responses at low versus high absorbed dose [24], [25]. Due to systemic regulation, these studies also illustrate the importance of using *in vivo* studies to elucidate biological responses to ionizing radiation. We have previously shown dose-dependent transcriptional responses highly associated with metabolism and stress-related processes in kidney tissue after ^177^Lu-octreotate administration [26]. Although DNA damage is considered to be a major molecular lesion following radiation exposure, responses to DNA damage were not detected in kidney tissue at the mRNA level. These studies further demonstrate the complex responses seen after radionuclide administration. Furthermore, kidney toxicity profiles after high absorbed dose exposure of ^177^Lu-octreotate administration (90–150 MBq injected activity) revealed morphological changes, elevated serum creatinine, and increased urea levels [4].

The aim of this study was to investigate the variation of the miRNA expression levels and their influence on the expression of miRNA target genes in normal renal cortical tissue after ^177^Lu-octreotate administration.

## Materials and Methods

### Animals and radiopharmaceutical preparation

The radiopharmaceuticals, ^177^LuCl_3_ and DOTA-Tyr^3^-octreotate, were purchased from I.D.B. Holland (Baarle-Nassau, Netherlands). Radiolabeling of DOTA-Tyr^3^-octreotate with ^177^LuCl_3_ was performed according to the manufacturer's instructions. A fraction of peptide bound ^177^Lu of >99% was considered adequate, as determined by instant thin layer chromatography. The ^177^Lu-octreotate stock solution was diluted to the final activity concentrations with saline solution.

The animal handling has previously been reported [26]. In brief, all animals had access to food and water *ad libitum*. Female BALB/c nude mice were divided into six groups containing three animals each and i.v. injected with 1.3, 3.6, 14, 45, or 140 MBq of ^177^Lu-octreotate through the tail vein. The control group was mock-treated with an i.v. injection of saline solution. The animals were killed by cardiac puncture under anesthesia (pentobarbitalnatrium, APL, Sweden) 24 h after injection. The kidneys were removed, flash frozen in liquid nitrogen, and stored at −80°C until further molecular studies. The experimental protocol was approved by the Ethical Committee on Animal Experiments in Gothenburg, Sweden.

### Dosimetry

The activity concentration was measured in one of the kidneys from each animal at the time of death using a gamma counter (Wallac Oy, Finland). The cumulated activity was estimated from the measured activity concentration together with data from a previously published biodistribution study of ^177^Lu-octreotate in BALB/c nude mice [27].

The absorbed dose to the kidneys was calculated according to the Medical Internal Radiation Dose (MIRD) formalism [28]:
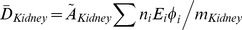
where Ã_Kidney_ is the cumulated activity during the first 24 h after injection, the product n_i_E_i_ is the average energy emitted by electrons per decay, 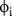 is the absorbed fraction (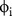 = 0.93 [29]), and m_Kidney_ is the mass of the kidney investigated. Only the electron contribution was included.

### Total RNA extraction and miRNA analysis

The renal cortical tissue was dissected from the flash-frozen kidneys and homogenized using the Mikro-Dismembrator S ball mill (Sartorius Stedim Biotech, Aubagne Cedex, France). Total RNA (including miRNA) was extracted using the miRNeasy Mini Kit (Qiagen, Hilden, Germany) according to the manufacturer's instructions. The RNA 6000 Nano Kit and the Small RNA kit were used with Agilent 2100 Bioanalyzer (Agilent Technologies, Santa Clara, CA, USA) to assess the RNA integrity and the presence of miRNA in the samples, respectively. RNA Integrity Number (RIN) values above 6.0 were accepted for further analysis.

The RNA samples were processed at the TATAA Biocenter (Gothenburg, Sweden). Labeling and hybridization were performed using the miRCURY microRNA Hy5 Power labeling kit (Exiqon A/S, Vedbaek, Denmark) and the Mouse miRNA oligo chip 4plex containing approximately 1,300 miRNAs (Toray Industries, Tokyo, Japan), according to the 3DGENE miRNA label Hybridization 4plex v1 protocol from Toray Industries, respectively. The chips were scanned with the Gene Scanner 3000 (Toray Industries, Tokyo, Japan) using standard settings for mouse miRNA v.19. Background subtraction and global normalization were performed using the Gene Scanner software.

Differentially regulated miRNAs were identified using two-tailed t-test between control and test samples (GenEx, Multid Analyses AB, Gothenburg, Sweden). miRNAs with *p*-values<0.05 and fold-change of >1.5 were considered significant. A target gene analysis was performed using statistically significant miRNAs together with previously published transcriptome data [26] with the IPA software (Ingenuity Systems, Redwood City, USA).

### Quantitative real-time PCR (QPCR)

Four differentially expressed miRNAs (miR-15a-5p, miR-92a-3p, miR-107, and miR-194-5p) were selected for verification by QPCR. Another eight constitutive miRNAs (let-7d-5p, let-7f-5p, miR-23a-3p, miR-26a-5p, miR-30a-3p, miR-3od-5p, miR-191-5p, and miR-192-5p), which showed homogeneous expression in the microarray analysis were used for normalization. The samples were reversely transcribed using the Universal cDNA Synthesis Kit II (Exiqon) according to the manufacturer's protocol. The correlation between the microarray and the QPCR results was estimated by the Pearson correlation coefficient.

## Results

In this study, we used an *in vivo* mouse model to identify differentially regulated miRNAs in renal cortical tissues following ^177^Lu-octreotate administration. The absorbed dose to the kidneys was determined to 0.13, 0.34, 1.3, 4.3, and 13 Gy 24 h after injection of 1.3, 3.6, 14, 45, and 140 MBq of ^177^Lu-octreotate, respectively. The absorbed dose estimated to infinity time would correspond to 0.31, 0.85, 3.3, 11, and 32 Gy, respectively.

**Figure 1:**
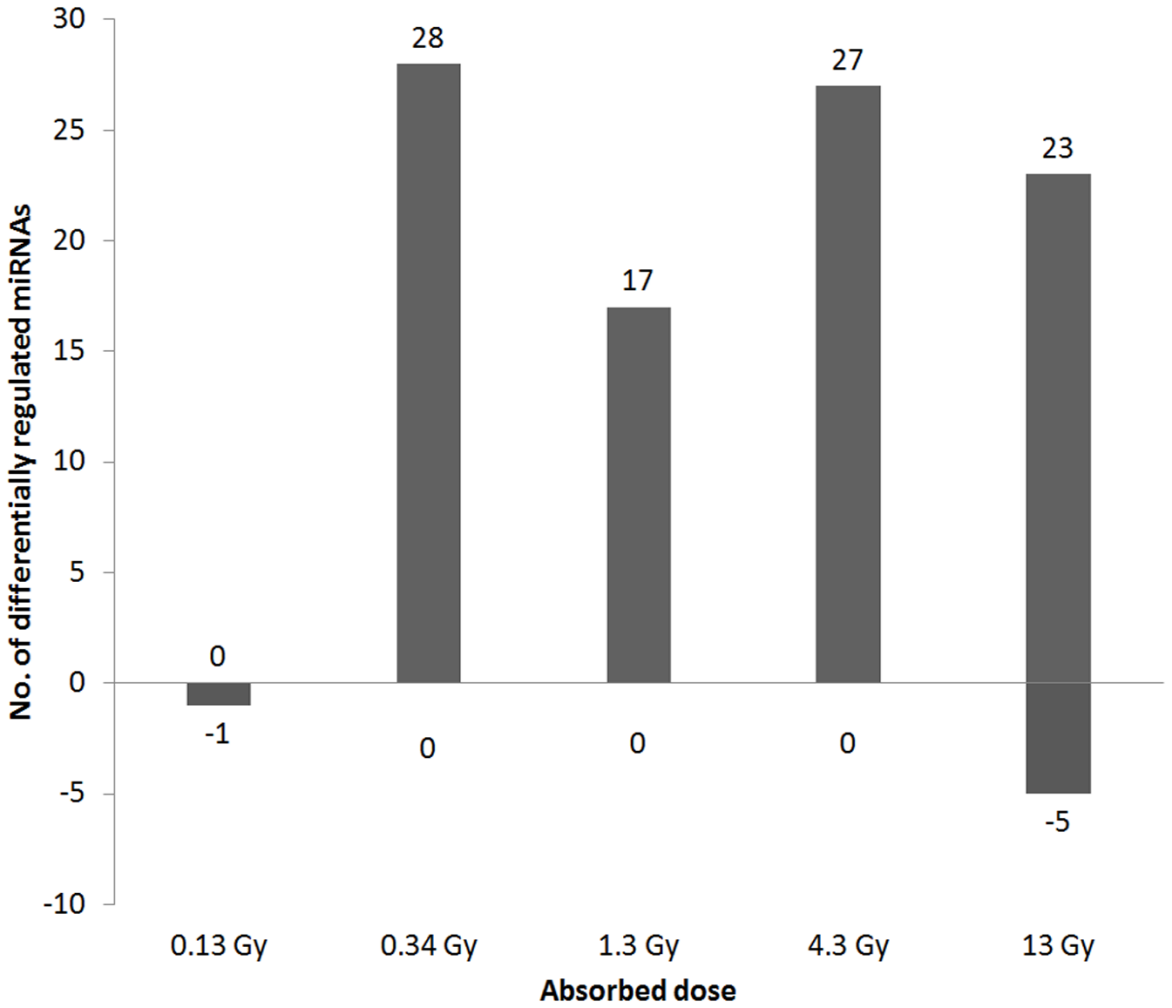
Number of differentially regulated miRNAs. Number of differentially regulated miRNAs in renal cortical tissue from mice 24 h after injection of different amounts of ^177^Lu-octreotate, resulting in absorbed dose to kidney of 0.13–13 Gy. Positive numbers indicate up-regulation, while negative numbers indicate down-regulation.

In total, 57 differentially regulated miRNAs were identified at the different dose levels, with 1, 29, 21, 27, and 31 differentially regulated miRNAs in the 0.13, 0.34, 1.3, 4.3, and 13 Gy groups, respectively (Figure 1). Few miRNAs were down-regulated after ^177^Lu-octreotate exposure. No down-regulated miRNAs were found at 0.34, 1.3, or 4.3 Gy, while only one miRNA (miR-365) was down-regulated at 0.13 Gy, and five miRNAs (let-7k, miR-690, miR-709, miR-1902, and miR-6239) were down-regulated at 13 Gy.

**Figure 2:**
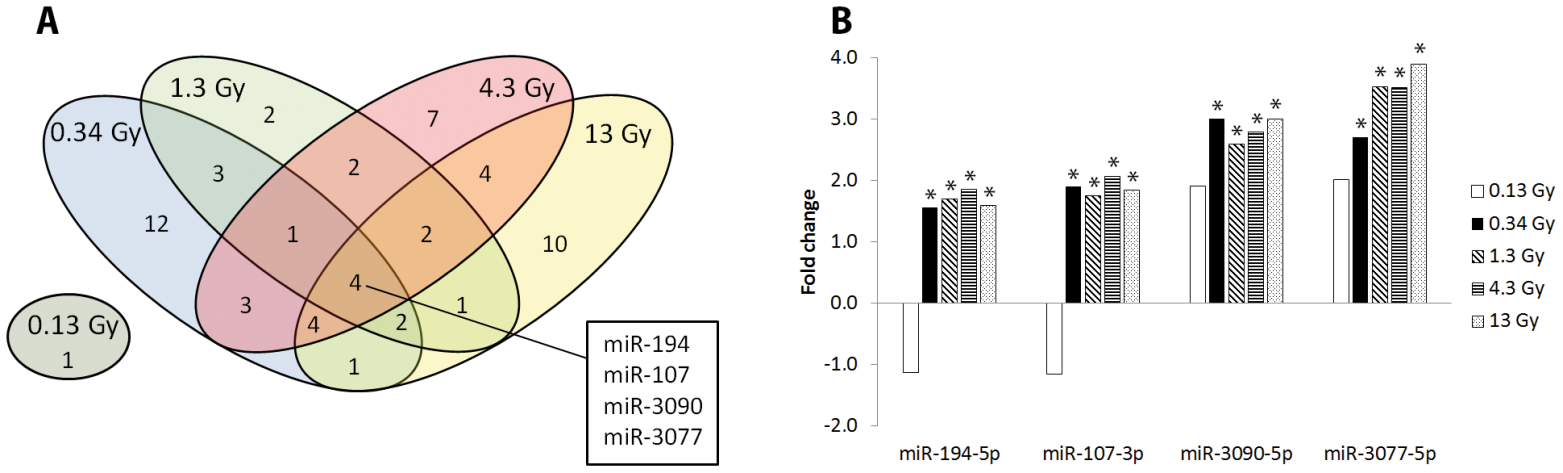
Distribution of differentially regulated miRNAs. (A) Distribution of differentially regulated miRNAs in mouse kidney cortex 24 h after ^177^Lu-octreotate administration (corresponding to absorbed dose to kidney of 0.13–13 Gy). The 0.13 Gy dose level did not share any regulated miRNAs with the other dose levels. Four miRNAs were shared between the groups receiving 0.34, 1.3, 4.3, and 13 Gy. The differential expression levels compared to control of these four miRNAs are shown in (B). * significant expression with *p*<0.05 and fold-change >1.5.

No miRNAs were differentially regulated at all absorbed doses (Figure 2). Interestingly, only miRNA (miR-365) was significantly regulated at 0.13 Gy, which was not found at the other dose levels. The expression level of the majority of the regulated miRNAs varied between the absorbed doses. However, four miRNAs (miR-194-5p, miR-107-3p, miR-3090-5p, and miR-3077-5p) were commonly and in general consistently regulated at 0.34, 1.3, 4.3, and 13 Gy (Figures 2A and 2B), with the exception of miR-3077 which showed a tendency towards increased expression level with increased absorbed dose. The intensity values of these miRNAs were found to generally deviate by 10–20% between the animals in the same exposure group, but higher deviations were occasionally observed.

The impact of microRNA regulation on mRNA transcriptional patterns was studied by integrating the miRNA and mRNA expression profiling data for renal cortical tissue from the same mice [26]. The expression levels (fold-change relative to the control) of the differentially regulated miRNAs are shown in Table 1, together with the predicted miRNA target genes. A strong negative correlation was found between the regulation of the target genes and the recurrently regulated miR-107 miRNA (the *Fam49a*, *Hmgcs2*, and *Slc25a10* genes (correlation <−0.70)). Similar results were found for the *Fga* and *Rnase6* genes as targets for miR-194. Furthermore, a positive correlation between the *Per1* gene and miR-194 (correlation >0.8) were found. The target prediction analysis revealed a strong association with immune response pathways. The antigen presentation pathway was affected at absorbed doses higher than 0.13 Gy, while interferon signaling was found at 0.34, 1.3, and 4.3 Gy (Table 2). p53 signaling was affected at the highest absorbed dose (13 Gy).

**Table 1:** Regulated miRNAs and their predicted target genes.

	0.13 Gy	0.34 Gy	1.3 Gy	4.5 Gy	13 Gy	Target Genes
**let-7e-5p**	-	2.17	-	-	-	*S100a8, Uhrf1*
		(0.033)				
**let-7k**	-	-	-	-	−1.75	*Cdkn1A, Krt19, S100a8, Scd, Zmat3*
					(0.039)	
**miR-10a-5p**	-	2.94	-	-	-	*Cyp24A1*
		(0.024)				
**miR-15a-5p**	-	-	1.74	2.05	2.37	*Ak4, Cd274, Irf1, Kcnk5, Phlda3, Pim3, Rab3D, Serpina10, Slc13A3, Slc9A8, Tmem43, Usp2, Zmat3, C1Qb, Cyp1B1, G0S2, Meox2, Parp14, Pdk4, Ppara*
			(0.018)	(0.002)	(0.001)	
**miR-15b-5p**	-	-	1.76	-	-	*C1Qb, Cd274, Cyp1B1, Irf1, Kcnk5, Parp14, Pdk4, Phlda3, Pim3, Psat1, Serpina10, Slc9A8*
			(0.042)			
**miR-17-5p**	-	2.14	-	-	-	*Ak4, Cd274, F2R, Irf1*
		(0.042)				
**miR-20b-5p**	-	2.10	-	2.04	-	
		(0.049)		(0.035)		
**miR-21a-5p**	-	-	-	-	1.74	*Acta2, Cdkn1A, Nlrc5, Per2, Slc12A1*
					(0.028)	
**miR-22-3p**	-	-	-	1.65	1.72	*Cdkn1A, Ciita, Ddit4, Phlda3, Slc25A10, Tp53Inp1, Cidec, Gabrb3, Gbp5, Nt5E, Ppara, Tap1*
				(0.005)	(0.011)	
**miR-26b-5p**	-	-	-	2.09	-	*C19Orf66, Gabrb3, Parp14, Pck1, Serpina10, Slc12A1, Slc25A20, Tap1, Tmem43*
				(0.009)		
**miR-30b-5p**	-	-	-	-	1.55	
					(0.041)	
**miR-30e-5p**	-	-	2.36	-	1.68	*Clec2D, Cyp24A1, Gldc, Per2, Slc9A8, Socs3, Stat1*
			(0.009)		(0.019)	
**miR-30c-5p**	-	-	-	2.05	1.78	*Clec2D, Cyp24A1, Ddit4, Fam49A, Gldc, Per2, Rab3D, Slc9A8, Tp53Inp1, Usp2, Marcks, Nt5E, Slc22A5, Socs3, Stat1, Vstm4, Zbtb7A*
				(0.012)	(0.004)	
**miR-92a-3p**	-	3.03	-	1.71	2.20	*Cdkn1A, Cldn11, Ddit4, Irf1, Paqr7, Per2*
		(0.003)		(0.046)	(0.008)	
**miR-99b-5p**	-	1.70	-	-	-	
		(0.049)				
**miR-106a-5p**	-	1.97	1.77	-	1.87	*Cd274, Cdkn1A, F2R, Irf1*
		(0.003)	(0.035)		(0.028)	
**miR-106b-5p**	-	-	-	2.18	2.01	*Ak4, Cd274, Cdkn1A, Irf1, Mmp2, Tp53Inp1, Zmat3, F2R, Ppara, Zbtb7A*
				(0.034)	(0.035)	
**miR-107-3p**	-	1.90	1.75	2.07	1.85	*Fam49A, Hmgcs2, Slc25A10, Slc9A8, Sypl2, C1Qb, Pdk4*
		(0.001)	(0.002)	(0.030)	(0.002)	
**miR-125a-5p**	-	1.97	1.53	-	-	*Acsm2A, Cyp11B2, Cyp24A1, Irf1, Ms4A6A, Nlrc5, Parp14, Pigr, Psmb8, Slc25A10, Usp2, Vstm4*
		(0.022)	(0.046)			
**miR-125b-5p**	-	1.84	-	-	-	
		(0.050)				
**miR-140-3p**	-	1.97	-	-	-	*Ak4, Cd274, Marcks*
		(0.011)	(0.014)			
**miR-151-5p**	-	2.34	1.58	-	-	*Laptm5, Tap1*
		(0.004)	(0.038)			
**miR-181a-5p**	-	2.02	-	-	2.16	*Ccng1, Ddit4, Fam49A, Rin2, Scd, Slc19A2, Slc25A25, Slc9A8, Tfrc, Egr1, Marcks, Socs3*
		(0.026)			(0.050)	
**miR-194-5p**	-	1.56	1.70	1.86	1.60	*Per1, Rnase6, Tfrc, Tmem43, Fga*
		(0.027)	(0.049)	(0.031)	(0.026)	
**miR-195a-5p**	-	-	-	-	2.26	
					(0.043)	
**miR-199b-5p**	-	2.21	2.39	-	2.38	*Clec2D, Glul, Pck1, Slc9A8*
		(0.008)	(0.023)		(0.014)	
**miR-199a-5p**	-	2.22	-	2.45	2.61	*Clec2D, Slc9A8, Pck1*
		(0.029)		(0.014)	(0.001)	
**miR-199a-3p**	-	1.73	-	-	-	
		(0.006)				
**miR-204-5p**	-	-	-	2.20	2.32	*Ak4, Arhgap30, Ms4A6A, Slc19A2, Tp53Inp1, Gabrb3, Zbtb7A*
				(0.021)	(0.029)	
**miR-214-3p**	-	2.90	-	2.78	-	*Afmid, C19Orf66, Gpd1, Parp14, Phlda3, Rnase6, Serpina1, Slc13A3, Slc25A10, Slc25A25, Slc25A42, Tmem43, Vstm4*
		(0.016)		(0.047)		
**miR-222-3p**	-	-	-	2.38	-	*Cldn11, Cyp1B1, Marcks, Mdm2, Socs3*
				(0.018)		
**miR-342-3p**	-	2.25	-	-	-	*Ak4, Gldc, Irf1, Slc22A5*
		(0.019)				
**miR-361-5p**	-	1.76	1.95	1.85	-	*Pdk4, Sucnr1, Zmat3, Mgarp*
		(0.044)	(0.022)	(0.046)		
**miR-365-3p**	−2.36	-	-	-	-	*Cyp24A1, Gbp5, Ms4A6A*
	(0.029)					
**miR-378c**	-	2.01	-	-	-	
		(0.018)				
**miR-378a-3p**	-	2.76	-	-	-	*Igfbp4, Uhrf1*
		(0.021)				
**miR-425-5p**	-	-	2.13	2.27	-	*Cd274, Gabrb3, Gbp5, Nlrc5, Slc25A20, Zmat3*
			(0.025)	(0.016)		
**miR-451a**	-	-	-	4.07	-	*Akr1B1, Meox2, Psmb8, Spc25, Zmat3*
				(0.048)		
**miR-484**	-	2.94	1.54	-	-	*Ccng1, Lpin1, Per1, Pim3, G0S2, Itih4, Usp2*
		(0.012)	(0.004)			
**miR-677-3p**	-	-	1.66	-	-	
			(0.006)		(0.047)	
**miR-690**	-	-	-	-	−1.81	
					(0.015)	
**miR-709**	-	-	-	-	−2.51	*Afmid, Ccng1, Ccrn4L, Cox6B2, Mdm2, Mreg, Nlrc5, Paqr7, Per2, Rab3D, Slc13A3, Slc19A2, Slc25A10, Tfrc, Tsc22D1*
					(0.003)	
**miR-1839-3p**	-	-	-	2.26	-	
				(0.048)		
**miR-1902**	-	-	-	-	−1.51	
					(0.035)	
**miR-2137**	-	-	-	3.42	-	
				(0.038)		
**miR-2861**	-	-	2.53	3.02	-	*Angptl4, Arhgap30, Cd274, Ciita, Cox6B2, Cryab, Cyp24A1, Hes6, Irf1, Kcnk5, Parp14, Pdk4, Phlda3, Plin4, Ppara, Serpina1, Slc25A25, Slc25A42, Socs3, Slc9A8*
			(0.037)	(0.035)		
**miR-3077-5p**	-	2.72	3.53	3.52	3.90	*Afmid*
		(0.027)	(0.005)	(0.004)	(0.008)	
**miR-3084-3p**	-	1.70	-	1.78	-	*Ccng1, Cldn11, Coro1A, Hes6, Irf1, Lpin1, Parp14, Sypl2, Tmem43, Tsc22D1, Zmat3*
		(0.003)		(0.003)		
**miR-3090-5p**	-	3.01	2.59	2.80	3.00	
		(0.002)	(0.010)	(0.005)	(0.013)	
**miR-3096b-3p**	-	-	-	-	2.58	
					(0.017)	
**miR-3102-5p**	-	2.31	-	2.84	2.08	*Aaas, Cd274, Coro1A, Cxcl9, Slc13A3, Serpina1*
		(0.024)		(0.027)	(0.038)	
**miR-5627-5p**	-	-	-	1.72	-	
				(0.044)		
**miR-6239**	-	-	-	-	−2.00	
					(0.019)	
**miR-6240**	-	-	-	2.70	-	*Laptm5, Meox2, Pck1, Zmat3*
				(0.003)		
**miR-6244**	-	2.85	-	3.66	2.83	
		(0.005)		(0.007)	(0.012)	
**miR-6402**	-	-	2.68	2.42	2.85	
			(0.014)	(0.037)	(0.027)	
**miR-6538**	-	-	-	-	2.39	
					(0.022)	

(-) indicates no change between the test and control samples.

Differentially regulated miRNAs and their predicted target genes according to IPA miRNA target filter analysis in renal cortical tissue from mice i.v. injected with different amounts of ^177^Lu-octreotate, resulting in an absorbed dose to kidney of 0.13–13 Gy 24 h after injection. The transcriptome data are taken from [26]. The numbers represent the fold-change in expression compared to unirradiated control and p-values are presented in parenthesis. Significant expression: *p*<0.05 and fold-change >1.5.

**Table 2:** Affected signaling pathways and upstream regulators.

Absorbed dose	Top pathways		Upstream regulator		
	Pathway	p-value	Gene	Molecule type*	Predicted activation state
**0.13 Gy**	**VDR/RXR Activation**	2.00E-02			
**0.34 Gy**	**Antigen Presentation Pathway**	1.12E-04	***Stat3***	transcription regulator	Inhibited
	**Interferon Signaling**	1.23E-04	***Ifng***	cytokine	Inhibited
	Circadian Rhythm Signaling	5.50E-03	***Tnf***	cytokine	Inhibited
	Coagulation System	6.17E-03	***Tlr3***	transmembrane receptor	Inhibited
	**LXR/RXR Activation**	6.17E-03			
**1.3 Gy**	**Antigen Presentation Pathway**	1.23E-04	**mir-21**	microRNA	Activated
	**Interferon Signaling**	1.38E-04	***SocsS1***	other	Activated
	**JAK/Stat Signaling**	1.66E-03	***Ifng***	cytokine	Inhibited
	Prolactin Signaling	2.09E-03	***Stat3***	transcription regulator	Inhibited
	**IL-22 Signaling**	3.16E-03			
**4.3 Gy**	**Antigen Presentation Pathway**	1.51E-05	***Ppara***	ligand-dependent nuclear receptor	Activated
	**Interferon Signaling**	1.74E-05	**mir-21**	microRNA	Activated
	Coagulation System	8.91E-04	***Tp73***	transcription regulator	Activated
	**JAK/Stat Signaling**	5.75E-03	***Pparg***	ligand-dependent nuclear receptor	Activated
	Prolactin Signaling	7.41E-03	***Tp53***	transcription regulator	Activated
			*Sparc*	other	Activated
			***Socs1***	other	Activated
			***Ifng***	cytokine	Inhibited
			***Stat1***	transcription regulator	Inhibited
			***Stat3***	transcription regulator	Inhibited
**13 Gy**	**Antigen Presentation Pathway**	2.14E-04	***Tp53***	transcription regulator	Activated
	**p53 Signaling**	5.13E-04	***Tp73***	transcription regulator	Activated
	HER-2 Signaling in Breast Cancer	3.89E-03			
	Agranulocyte Adhesion and Diapedesis	5.01E-03			
	Bladder Cancer Signaling	5.50E-03			

*according to IPA.

Top five affected signaling pathways and predicted activation/inhibition of upstream regulators in renal cortical tissue from mice i.v. injected with different amounts of ^177^Lu-octreotate, resulting in absorbed dose to the kidney of 0.13–13 Gy 24 h after injection. Pathways and upstream regulators which are associated with immune response are highlighted in bold.

The majority of affected upstream regulators were transcription factors and cytokines, among which only one miRNA upstream regulator (miR-21) was affected. An analysis of the predicted activation state of upstream regulators was also conducted with the IPA software (Table 2). The highest number of affected upstream regulators was found at 4.3 Gy. The predicted activation/inhibition of upstream regulators corresponded well to the pathways affected, e.g. IFNG inhibition at 0.34, 1.3, and 4.3 Gy corresponding to interferon signaling in the pathway analysis. Additionally, *TP53* and *TP73* activation at 13 Gy corresponded to p53 signaling.

Among the 57 differentially regulated miRNAs identified in the present study, 39 have been previously reported to be responsive to ionizing radiation exposure (Table S1 and Table 3). These studies include investigations of the response to different types of ionizing radiation (e.g. gamma, x-rays, and Fe-56 ions) in different cell lines, but also tissues from irradiated mice and cancer patients. Several miRNA families were found to be commonly regulated with respect to absorbed dose, time after irradiation, as well as cell line/tissue. The differential regulation of members of the let-7 family was reported in 14 studies, with absorbed doses ranging between 0.05 and 19 Gy in a wide variety of cell lines and tissues. In addition, the miR-10, 15, 17, and 181 families were similarly regulated. The remaining eighteen differentially regulated miRNAs identified in the present study (miR-484, miR-677-3p, miR-690, miR-709, miR-1839-3p, miR-1902, miR-2137, miR-3077-5p, miR-3084-3p, miR-3090-5p, miR-3096b-3p, miR-3102-5p, miR-5627-5p, miR-6239, miR-6240, miR-6244, miR-6402, and miR-6538) have not previously been associated with ionizing radiation.

**Table 3:** Radiation-responsive miRNAs.

miRNA family	miRNA	Present study	Previous studies			
			Absorbed dose	Time	Radiation Source	Cell line/Tissue
**let-7**	let-7 a–k	0.34, 13 Gy	0.05, 0.2, 0.25, 0.5, 1, 1.25, 2, 2.5, 3, 5, 6, 8, 10, 18.8 Gy	0.5, 1, 2, 3, 4, 6, 8, 24, 48, 96, 168 h	Co-60, gamma, Cs-137, 90 kV x-ray, 6 MV gamma, 6 MV photon	NorHuFib, CRL2741, human 3D tissue system, A549, B lymphoblasts IM9, Male mouse spleen, differentiated kerotinocytes, Normal thyroid cells, human PBL, CD34+, human blood (TBI), Female mouse frontal lobe, endothelial cells, hFOB, U87MG glioblastoma
**miR-10**	miR-10, miR-99, miR-100, miR-125	0.34, 1.3 Gy	0.1, 0.25, 0.5, 1, 2, 2.5, 3, 5, 6, 8, 10 Gy	0.5, 1, 3, 6, 8, 48, 168 h	Cs-137, Co-60, 6 MV x-ray, gamma, proton, Fe-56	Mouse blood, B lymphoblasts IM9, proliferating kerotinocytes, NorHuFib, endothelial cells, human 3D tissue system, human PBL, hFOB, Female mouse hippocampus, CRL2741, differentiated kerotinocytes, A549, LNCaP prostate cancer cell
**miR-15**	miR-15, miR-16, miR-195	1.3, 4.3, 13 Gy	0.05, 0.2, 0.25, 0.5, 1, 1.25, 2.5, 3, 5, 6, 8, 10, 20, 40 Gy	0.5, 1, 2, 3, 4, 6, 8, 24, 48, 168 h	gamma, 90 kV x-ray, Co-60, Cs-137, 6 MV gamma	CRL2741, A549, Female mouse cerebellum, NorHuFib, human 3D tissue system, hFOB, B lymphoblasts IM9, endothelial cells, lung carcinoma cell A549, human blood (TBI), human PBL, CD34+, differentiated kerotinocytes
**miR-17**	miR-17, miR-18 a,b, miR-20 a,b, miR-93, miR-106 a,b	0.34, 1.3, 4.3, 13 Gy	0.05, 0.2, 1, 1.25, 2, 2.5, 6, 10, 20, 40 Gy	0.5, 2, 3, 4, 6, 8, 24, 48, 96, 168 h	gamma, Cs-137, 90 kV x-ray, 6 MV gamma, 250 keV x-rays, 15 MeV photons	human 3D tissue system, human blood (TBI), human PBL, Male mouse cerebellum, proliferating kerotinocytes, CRL2741, A549, B lymphoblasts IM9, endothelial cells, foreskin fibroblasts, Male mouse spleen, Female mouse hippocampus, Normal thyroid cells, Male mouse frontal lobe, lung carcinoma cell A549, prostate cancer cells LNCaP
**miR-21**	miR-21	13 Gy	0.05, 0.2, 0.25, 0.5, 1, 1.25, 2, 2.5, 3, 5, 10 Gy	1, 2, 4, 6, 8, 24, 168 h	Co-60, gamma, 6 MV gamma, Cs-137	NorHuFib, human 3D tissue system, endothelial cells, A549, human blood (TBI), B lymphoblasts IM9, human PBL
**miR-22**	miR-22	4.3, 13 Gy	0.2, 1, 2, 2.5, 6, 18.8 40 Gy	0.5, 2, 4, 6, 8, 24, 168 h	gamma, Cs-137, 15 MeV photons, 6 MV photon, 90 kV x-ray	human 3D tissue system, lung carcinoma cell A549, prostate cancer cells LNCaP, CRL2741, A549, U87MG glioblastoma, Female mouse frontal lobe
**miR-25**	miR-25, miR-92 a,b	0.34, 4.3, 13 Gy	0.2, 1, 2, 2.5 Gy	0.5, 1, 2, 8, 48, 96 h	250 keV x-rays, 90 kV x-rays, gamma	human 3D tissue system, CRL2741, A549, Female mouse frontal lobe, CD34+, foreskin fibroblasts
**miR-26**	miR-26 a,b	4.3 Gy	0.25, 0.2, 0.5, 1, 1.25, 2, 2.5, 3, 5, 6, 10 Gy	0.5, 1, 2, 3, 4, 24, 48, 168 h	gamma, Cs-137, Co-60	human 3D tissue system, A549, proliferating kerotinocytes, NorHuFib, CRL2741, human blood (TBI)
**miR-28**	miR-28 a,c, miR-151 a,b	0.34, 1.3 Gy	0.2, 1, 2, 2.5, 5, 6, 8 Gy	1, 2, 3, 24, 48, 96, 168 h	90 kV x-ray, Cs-137, gamma	A549, differentiated kerotinocytes, Female mouse hippocampus, hFOB, Mouse blood, human PBL, human 3D tissue system
**miR-30**	miR-30 a–e	1.3, 4.3, 13 Gy	0.2, 2, 2.5, 6, 8, 40 Gy	0.5, 1, 2, 8, 24, 48, 168 h	gamma, 15 MeV photons, Cs-137	human 3D tissue system, prostate cancer cells LNCaP, CRL2741, A549, lung carcinoma cell A549, CD34+, hFOB, human PBL
**miR-107**	miR-103 a,b, miR-107	0.34, 1.3, 4.3, 13 Gy	0.2, 1, 1.25, 2, 6, 18.8 Gy	0.5, 1, 4, 24, 48, 168 h	gamma, Cs-137, 6 MV photons	human 3D tissue system, CD34+, human blood (TBI), B lymphoblasts IM9, LNCaP prostate cancer cell, U87MG glioblastoma
**miR-140**	miR-140	0.34 Gy	1, 1.25 Gy	4, 6 h	90 kV x-ray, gamma	Female mouse frontal lobe, human blood (TBI)
**miR-181**	miR-181 a–d	0.34, 13 Gy	0.2, 1, 2, 2.5, 6, 10, 18.8 Gy	0.5, 2, 3, 4, 6, 8, 24, 96, 168 h	gamma, 6 MV photon, Cs-137, 250 keV x-rays, 90 kV x-ray	human 3D tissue system, A549, U87MG glioblastoma, human PBL, differentiated kerotinocytes, foreskin fibroblasts, Female mouse spleen, Normal thyroid cells, Male mouse hippocampus, CRL2741, Female mouse cerebellum
**miR-194**	miR-194	0.34, 1.3, 4.3, 13 Gy	1, 2.5 Gy	2, 6, 24 h	90 kV x-ray, gamma	Female mouse frontal lobe, A549
**miR-199**	miR-199 a,b	0.34, 1.3, 4.3, 13 Gy	0.05, 0.2, 1.25, 2, 6, 10 Gy	2, 8, 24 h	15 MeV photons, Cs-137, gamma	prostate cancer cells LNCaP, B lymphoblasts IM9, human blood (TBI), human PBL
**miR-204**	miR-204, miR-211	4.3, 13 Gy	0.5, 1, 5 Gy	6, 24, 96 h	proton, 90 kV x-ray, Cs-137, Fe-56	mouse blood, Female mouse cerebellum, Female mouse hippocampus
**miR-214**	miR-214, miR-3120	0.34, 4.3 Gy	1, 2.5 Gy	2, 6, 8, 24 h	90 kV x-ray, gamma	Male mouse hippocampus, A549
**mir-221**	miR-221, miR-222	4.3 Gy	0.2, 0.25, 0.5, 1, 1.25, 2, 2.5, 3, 5, 10 Gy	0.5, 1, 2, 4, 6, 8, 24, 48, 96, 168 h	gamma, 90 kV x-ray, Cs-137, Co-60	human 3D tissue system, CRL2741, A549, human blood (TBI), Male mouse spleen, human PBL, NorHuFib, Female mouse cerebellum
**miR-342**	miR-342	0.34 Gy	0.01, 0.5, 5, 6, 10 Gy	3, 24 h	Cs-137, Fe-56	B lymphoblasts IM9, differentiated kerotinocytes, proliferating kerotinocytes, Mouse blood
**miR-361**	miR-361	0.34, 1.3, 4.3 Gy	2.5 Gy	2, 8, 24 h	gamma	CRL2741, A549
**miR-365**	miR-365 a,b	0.13 Gy	1, 2.5, 10 Gy	2, 8, 24 h	Cs-137, gamma	Normal thyroid cells, A549, B lymphoblasts IM9
**miR-378**	miR-378 a–e	0.34 Gy	0.2, 2 Gy	4 h	Cs-137	human PBL
**miR-425**	miR-425	1.3, 4.3 Gy	0.2, 2 Gy	0.5, 4, 48, 168 h	Cs-137, gamma	human 3D tissue system, human PBL
**miR-451**	miR-451 a,b	4.3 Gy	1, 18.8 Gy	4, 6 h	90 kV x-ray, 6 MV photon	Female mouse frontal lobe, U87MG glioblastoma
**miR-2861**	miR-2861	1.3, 4.3 Gy	2 Gy	1 h	gamma	CD34+

Differentially regulated miRNAs in the present study which have previously been associated with exposure to ionizing radiation. The miRNAs are categorized into miRNA families according to the microRNA database miRBase (www.mirbase.org). For each family, a summary of at which absorbed doses and times after irradiation this family has previously been found affected, together with radiation source and in which cell line/tissue it has been studied. A complete list with references are presented in Table S1.

In order to validate the microarray results, several miRNAs were selected for qRT-PCR analysis. The correlation between the microarray and qRT-PCR analysis was high. The miRNAs miR-15a-5p (r = 0.92), miR-107 (r = 0.91), and miR-194-5p (r = 0.72) showed a strong correlation whereas the miR-92a-3p (r = −0.11) revealed no correlation.

## Discussion

The kidneys are one of the major sites of side effects of ^177^Lu-octreotate therapy of neuroendocrine tumors due to the high uptake of the radiopharmaceutical in this organ [3], [5], [30]–[32]. Basic insight into how normal kidney tissue responds to ^177^Lu-octreotate exposure is therefore vital for the continued optimization of therapy with this radiopharmaceutical. In the present study, aberrant miRNA expression patterns were investigated in mouse renal cortical tissue following ^177^Lu-octreotate administration. Our findings demonstrate common miRNA deregulation among all the studied absorbed doses as well as dose-specific miRNA deregulation. miRNA families previously reported to be frequently associated with radiation exposure were detected. The results showed that exposure produced a strong immunological response on renal cortical tissue.

Kidney cortical tissue should be given extra attention because ^177^Lu-octreotate uptake takes place to a somewhat higher extent in this part of the kidney by, e.g., receptor-mediated endocytosis via the megalin/cubulin complex and somatostatin receptors, amino acid/oligopeptide transporters, pinocytosis, and passive diffusion [3], [5], [32]. Furthermore, it has previously been shown that the radioactivity localizes in the proximal tubules of the cortex, with less activity in the distal tubules and glomeruli, as evaluated by micro-autoradiography [3], and ^177^Lu-octreotate induced damage has been found in the cortical tissue [4]. Despite the differences seen in activity distribution and induced injury between the kidney cortex and medulla, the transcriptional data following ^177^Lu exposure show high similarities between the two tissues [26], [33]. However, discrepancies were found and include a stronger association with stress responses in the kidney cortex compared with kidney medulla following ^177^Lu-octreotate administration, further verifying the importance of studying the kidney cortical tissue response following exposure to this radiopharmaceutical.

The use of microarray analysis to investigate the miRNA response allows for a comprehensive view of the biological effects following exposure. However, care must be taken when interpreting these results and comparing them with other studies using alternative methods. A previous study has described the poor reproducibility between microarray and miRNA-seq data when miRNAs were associated with overall survival in ovarian cancer profiles [34]. A comparison between Agilent miRNA microarray and quantitative qRT-PCR measurements have, however, shown excellent correlation [35]. In the present study, a strong correlation between the microarray and the qRT-PCR results was found for the miRNAs miR-15a-5p, miR-107, and miR-194-5p. No correlation was found for the miR-92a-3p, indicating the importance of the verification of microarray results.

Few studies have investigated the miRNA response after ionizing radiation exposure, and these studies have primarily been performed using external irradiation on different cell lines. Furthermore, few studies have performed investigations using similar experimental setups. The differences in type of radiation, *in vitro*/*in vivo*, cell type, organ, dose, dose-rate, time after exposure etc., make comparisons between studies difficult. In the present study, the number of differentially regulated miRNAs was relatively constant among the highest absorbed doses (0.34–13 Gy), ranging from 17 to 28, whereas only miR-365 was differentially regulated at the lowest absorbed dose (0.13 Gy). These findings are in agreement with recent studies showing dose-dependent changes in miRNA expression levels, e.g., Templin *et al.* who reported a higher number of differentially regulated miRNAs in blood samples collected from mice exposed to 1 Gy compared with 0.5 Gy from 600 MeV protons (19 *vs.* 5 regulated miRNAs at 6 h and 6 *vs.* 3 regulated miRNAs at 24 h after irradiation) [36]. Dose-dependent miRNA regulation was also found in proliferating keratinocytes *in vitro*, where exposure to 10 mGy (^137^Cs) revealed fewer regulated miRNAs compared with exposure to 6 Gy 3 h after irradiation (2 *vs.* 8 regulated miRNAs) [37]. Furthermore, the lack of a dose-response in the number of regulated transcripts at doses higher than 0.13 Gy could be expected. This tendency has previously been shown at the transcriptional level after radionuclide administration [23]–[26], [33]. An explanation could be that the biological response is not facilitated by the number of miRNAs/transcripts regulated but instead of which miRNAs/transcripts that are regulated. With increased dose, an increased association towards stress response is observed, with both increased association towards immune response and inflammation [26], and we assume that if a dose-response is seen, it is in either individually regulated miRNAs/transcripts or more likely a panel of regulated miRNAs/transcripts.

The time aspect has also been proven to be an important model parameter during miRNA expression analysis. Several studies reported a time-dependent regulation of miRNAs, especially at lower absorbed doses [36], [38], [39]. Weidhaas *et al.* found that in the A549 lung cancer cell line, the majority of regulated miRNAs were detected 2 h after gamma exposure (2.5 Gy) and most of these returned to baseline levels 24 h after irradiation [38]. This decrease in the number of regulated miRNAs with time may be due to repair of radiation damage, where lower absorbed doses induce less damage [25].

In the present investigation, the majority of differentially regulated miRNAs were primarily up-regulated with only one miRNA down-regulated at 0.13 Gy (miR-365) and five down-regulated at 13 Gy (miR-709, miR-6239, miR-690, let-7k, and miR-1902); no down-regulated miRNAs were found at the intermediate dose levels (0.34–4.3 Gy). This finding is in contrast to results by Templin *et al.*, who found a higher number of down-regulated miRNAs after proton irradiation [36]. Furthermore, we were not able to identify any commonly regulated miRNAs at all absorbed doses. However, four miRNAs (miR-194, miR-107, miR-3090, and miR-3077) were commonly regulated at the highest four absorbed doses. Previous reports concluded that miRNA regulation is highly dependent on the irradiation parameters, e.g. radiation dose and time-point, in addition to cell type [36], [37], [40], [41].

Previously, we have observed that changes in mRNA expression in the kidney were evident after irradiation [26]. In the present investigation, the identified miRNAs are tissue specific and radiation induced. The majority of the identified radiation induced miRNAs are not evolutionary conserved because radiation is not a selection factor during evolution. Concerning the present study, an association with stress-related responses, e.g. antigen presentation pathway and interferon signaling, were found for the majority of the dose levels investigated. Components of the p53 signaling pathway were regulated at 13 Gy, predicting activation of upstream regulators *TP53* and *TP73*. A previous paper showed that the miR-34 family, which is a direct transcriptional target of p53, might induce cell cycle progression [42]. miR-34 has been reported to be differentially regulated after ionizing radiation in different cell lines, as well as in mouse spleen and brain, indicating the importance of this miRNA gene family in the response to ionizing radiation. In the present investigation, differential expression of miR-34 was not observed. This finding does not, however, show that p53 signaling pathway is not affected. It may still be affected by other activation mechanisms via other miRNAs. Furthermore, Simone *et al.* showed that miRNAs affected by exposure to ionizing radiation also showed differential regulation after exposure to H_2_O_2_ and etoposide [7]. This could indicate putative common miRNA signatures in response to genotoxic agents.

The majority of regulated miRNAs found in the present study have previously been detected after irradiation of a variety of cell lines and tissues with ionizing radiation (Table S1 and Table 3). The let-7 miRNA family are involved in cell proliferation, differentiation and inhibition of the Ras pathway [42], [43] and is one of the most commonly regulated miRNA family affected by ionizing radiation [11], [43]–[45]. The let-7 family has been found to be regulated at 0.05–19 Gy in a variety of cell lines and time points (0.5–168 h), and regulation of the let-7 family has also been regulated after chronic exposure to low dose ionizing radiation (3 mGy/h, 1 Gy) [46].

Other miRNA families that are commonly regulated after ionizing radiation include the miR-10, 15, 17, and 181 families. The miR-10 family includes four miRNAs (miR-10, 99, 100, and 125). miR-99 is involved in DNA damage response in breast and prostate cancer cells, where overexpression showed an adverse effect on the efficiency of DNA damage repair by both NHEJ and homologous recombination [47]. Overexpression of miR-100 can increase radiosensitivity in human glioma cells [21], while radioprotective effects have been observed in epithelial cells after miR-125a regulation [48]. The miR-15 family, including the miR-15, 16, and 195 miRNAs, induce apoptosis by targeting BCL2 [49] and are direct transcriptional targets of E2F1 which is involved in cell cycle control [50]. The miR-17 family (miR-17, 18, 20, 93, and 106) affect G1 checkpoint regulation, and overexpression in cancers activates cell proliferation, while the miR-181 family affect radiation sensitivity in certain cell lines [51]. Taken together, these miRNA families play an important role in cell stress responses, DNA repair, apoptosis and cell cycle control and members of these families were found consistently differentially regulated in the present study.

Eighteen miRNAs found in the present study have not previously been associated with ionizing radiation, i.e. miR-484, miR-677-3p, miR-690, miR-709, miR-1839-3p, miR-1902, miR-2137, miR-3077-5p, miR-3084-3p, miR-3090-5p, miR-3096b-3p, miR-3102-5p, miR-5627-5p, miR-6239, miR-6240, miR-6244, miR-6402, and miR-6538. An explanation could be that these miRNAs may be renal cortical tissue-specific. Two of the four miRNAs (miR-3077 and miR-3090) which were commonly regulated at the four highest absorbed doses were among these 18 miRNAs. To our knowledge no similar study has been performed on kidney tissue. Thus, further studies are warranted using larger sets of experimental assays to evaluate the role ionizing radiation plays on the function of these miRNAs in relation to tissue type, exposure conditions, time, and absorbed dose.

## Conclusion

In this study, distinct miRNA expression profiles were identified in mouse renal cortical tissue after administration of ^177^Lu-octreotate. The biological response observed revealed pronounced effects on stress response pathways and p53 signaling at the highest absorbed dose investigated (13 Gy). The miRNA signatures were in general dose-specific. In some instances there was a correlation between miRNA expression levels and the expression levels of miRNA target genes. Furthermore, a number of miRNAs were recurrently differentially expressed (miR-194-5p, miR-107-3p, miR-3090-5p, and miR-3077-5p). However, more research is needed to evaluate the role of these and other miRNAs in the kidneys in response to radionuclide administration.

## Supporting Information

Table S1 miRNAs responsive to ionizing radiation.
(PDF)
