# Supplementary material for: Distinct microRNA Expression Profiles in Mouse Renal Cortical Tissue after 177Lu-octreotate Administration
Source: PLoS One. 2014 Nov 11;9(11):e112645. doi: 10.1371/journal.pone.0112645 (PMC4227842; doi:10.1371/journal.pone.0112645)
Supplement: Table S1 — miRNAs responsive to ionizing radiation. (PDF) [file pone.0112645.s001.pdf]

Table S1. Radiation-responsive miRNAs

miRNAs which have previously been found to be regulated after exposure to ionizing radiation. Also presented is at which absorbed doses and times after irradiation these miRNAs have previously been found affected, together with radiation source and in which cell line/tissue it has been studied as well as its regulation. Complete reference list are presented at bottom of table

miRNA	Absorbed dose	Time	Radiation source	Cell line/Tissue	Regulation	Reference
let-7a	0.25, 0.5, 1, 3, 5, 10Gy	1h	Co-60	NorHuFib	-	Simone, Soule 2009
let-7a	0.2, 2Gy	0.5, 48, 168h	gamma	human 3D tissue system	+-	Dickey, Zemp 2011
let-7a	2.5Gy	2, 8, 24h	gamma	CRL2741	-	Weidhaas, Babar 2007
let-7a	2.5Gy	2, 8, 24h	gamma	A549	-	Weidhaas, Babar 2007
let-7a	1, 10Gy	24h	Cs-137	B lymphoblasts IM9	-	Cha, Shin 2009
let-7a	2.5Gy	6h	90 kV x-ray	Male mouse spleen	+	Ilnytskyy, Zemp 2008
let-7b	0.25, 0.5, 1, 3, 5, 10Gy	1h	Co-60	NorHuFib	-	Simone, Soule 2009
let-7b	0.2, 2Gy	0.5, 48h	gamma	human 3D tissue system	-	Dickey, Zemp 2011
let-7b	2.5Gy	2, 8, 24h	gamma	CRL2741	-	Weidhaas, Babar 2007
let-7b	2.5Gy	2, 8, 24h	gamma	A549	-	Weidhaas, Babar 2007
let-7b-5p	6Gy	3h	Cs-137	differentiated kerotinocytes	+	Joly-Tonetti, Vinuelas
let-7c	0.2, 2Gy	0.5, 48, 168h	gamma	human 3D tissue system	-	Dickey, Zemp 2011
let-7c	1, 10Gy	4, 24h	Cs-137	Normal thyroid cells	+	Nikiforova, Gandhi 2011
let-7c	2.5Gy	2, 8, 24h	gamma	CRL2741	-	Weidhaas, Babar 2007
let-7c	2.5Gy	2, 8, 24h	gamma	A549	-	Weidhaas, Babar 2007
let-7c	1Gy	24h	Cs-137	B lymphoblasts IM9	-	Cha, Shin 2009
let-7c	10 Gy	5, 15, 40h	gamma	PC3	-	Leung, Li 2014
let-7d	0.25, 0.5, 1, 3, 5, 10Gy	1h	Co-60	NorHuFib	+	Simone, Soule 2009
let-7d	0.2, 2Gy	0.5, 48, 168h	gamma	human 3D tissue system	-	Dickey, Zemp 2011
let-7d	1, 10Gy	4, 24h	Cs-137	Normal thyroid cells	+	Nikiforova, Gandhi 2011
let-7d	2.5Gy	2, 8, 24h	gamma	CRL2741	-	Weidhaas, Babar 2007
let-7d	2.5Gy	2, 8h	gamma	A549	-	Weidhaas, Babar 2007
let-7d	2.5Gy	6h	90 kV x-ray	Male mouse spleen	+	Ilnytskyy, Zemp 2008
let-7d	2 Gy	4h	proton	Mouse testis	+	Khan, Tariq 2013
let-7d-5p	10 Gy	5, 15, 40h	gamma	PC3	-	Leung, Li 2014
let-7e	0.25, 0.5, 1, 3, 5, 10Gy	1h	Co-60	NorHuFib	+	Simone, Soule 2009
let-7e	0.2, 2Gy	0.5, 48, 168h	gamma	human 3D tissue system	-	Dickey, Zemp 2011

let-7e	2.5Gy	2, 8, 24h	gamma	CRL2741	-	Weidhaas, Babar 2007
let-7e	2.5Gy	2, 8, 24h	gamma	A549	-	Weidhaas, Babar 2007
let-7e	10Gy	24h	Cs-137	B lymphoblasts IM9	-	Cha, Shin 2009
let-7e	0.2Gy	4h	Cs-137	human PBL	-	Girardi, De Pitta 2012
let-7e-5p	10 Gy	5, 15, 40h	gamma	PC3	-	Leung, Li 2014
let-7-5p	0.34Gy	24h	^{177}Lu-octreotate	mouse renal cortical tissue	+	present study
let-7f	0.2, 2Gy	0.5, 48, 168h	gamma	human 3D tissue system	-	Dickey, Zemp 2011
let-7f	1, 10Gy	4, 24h	Cs-137	Normal thyroid cells	-	Nikiforova, Gandhi 2011
let-7f	2Gy	1h	gamma	CD34+	+	Li, Ha 2012
let-7f	2.5Gy	2, 8, 24h	gamma	CRL2741	-	Weidhaas, Babar 2007
let-7f	2.5Gy	2, 8, 24h	gamma	A549	-	Weidhaas, Babar 2007
let-7f	1.25Gy	4h	gamma	human blood (TBI)	+	Templin, Paul 2011
let-7f	1Gy	24h	Cs-137	B lymphoblasts IM9	-	Cha, Shin 2009
let-7f	1Gy	96h	90 kV x-ray	Female mouse frontal lobe	+	Koturbash, Zemp
let-7f-2	0.05Gy	8h	Cs-137	B lymphoblasts IM9	-	Cha, Seong 2009
let-7g	0.25, 0.5, 1, 3, 5, 10Gy	1h	Co-60	NorHuFib	+	Simone, Soule 2009
let-7g	0.2, 2Gy	0.5, 168h	gamma	human 3D tissue system	-	Dickey, Zemp 2011
let-7g	2 Gy	6h	6MV gamma	endoth cells	+	Wagner-Ecker, Schwager 2010
let-7g	1, 10Gy	4, 24h	Cs-137	Normal thyroid cells	+-	Nikiforova, Gandhi 2011
let-7g	8Gy	1h	gamma	hFOB	+	Li, Ha 2012
let-7g	2.5Gy	24h	gamma	A549	+	Weidhaas, Babar 2007
let-7g	1.25Gy	4h	gamma	human blood (TBI)	+	Templin, Paul 2011
let-7g	18.8Gy	4h	6 MV photon	U87MG glioblastoma	+	Chen, Zhu 2009
let-7g	2.5Gy	6h	90 kV x-ray	Male mouse spleen	-	Ilnytskyy, Zemp 2008
let-7i	0.25, 0.5, 1, 3, 5, 10Gy	1h	Co-60	NorHuFib	+	Simone, Soule 2009
let-7i	0.2, 2Gy	0.5, 48h	gamma	human 3D tissue system	+-	Dickey, Zemp 2011
let-7i	2.5Gy	2, 8, 24h	gamma	A549	-	Weidhaas, Babar 2007
let-7k	13Gy	24h	^{177}Lu-octreotate	mouse renal cortical tissue	-	present study
miR-1	1.0 Gy	6 h	proton	mouse blood	-	Templin, Young 2012
miR-1	1Gy	4, 24h	Cs-137	Normal thyroid cells	-	Nikiforova, Gandhi 2011
miR-1	10Gy	4, 24h	Cs-137	Normal thyroid cells	-	Nikiforova, Gandhi 2011
miR-1	1Gy	96h	90 kV x-ray	Female mouse hippocampus	-	Koturbash, Zemp
miR-7	2.5Gy	24h	gamma	CRL2741	-	Weidhaas, Babar 2007

miR-7	2.5Gy	2h	gamma	A549	+	Weidhaas, Babar 2007
miR-7	8Gy	6h	gamma	HeLa	+	Hu, Tie 2013
miR-7	2.5Gy	6h	90 kV x-ray	Male mouse spleen	-	Ilnytskyy, Zemp 2008
miR-9-1	6Gy	24h	15 MeV photons	prostate cancer cells LNCaP	+	Li, Shi 2011
miR-9-3p	2.5Gy	8, 24 h	gamma	CRL2741	+-	Weidhaas, Babar 2007
miR-9-3p	18.8Gy	4h	6 MV photon	U87MG glioblastoma	+	Chen, Zhu 2009
miR-9-5p	10 Gy	5, 15, 40h	gamma	PC3	+	Leung, Li 2014
miR-10a	5Gy	6h	Cs-137	Mouse blood	+	Templin, Amundson 2011
miR-10a	2Gy	24h	Cs-137	human PBL	-	Girardi, De Pitta 2012
miR-10a-5p	0.34Gy	24h	^{177}Lu-octreotate	mouse renal cortical tissue	+	present study
miR-10b	0.5, 1Gy	6 h	p	mouse blood	-	Templin, Young 2012
miR-10b	2 Gy	4h	proton	Mouse liver	-	Khan, Tariq 2013
miR-15a	2.5Gy	2, 8, 24h	gamma	CRL2741	-	Weidhaas, Babar 2007
miR-15a	2.5Gy	2, 8, 24h	gamma	A549	-	Weidhaas, Babar 2007
miR-15a	8Gy	6h	gamma	HeLa	-	Hu, Tie 2013
miR-15a	1Gy	6h	90 kV x-ray	Female mouse cerebellum	+	Koturbash, Zemp
miR-15a-5p	1.3, 1.3, 13Gy	24h	^{177}Lu-octreotate	mouse renal cortical tissue	+	present study
miR-15a-5p	10 Gy	5, 15, 40h	gamma	PC3	-	Leung, Li 2014
miR-15b	0.250.5, 1, 3, 5, 10 Gy	1h	Co-60	NorHuFib	+	Simone, Soule 2009
miR-15b	0.2Gy	0.5h	gamma	human 3D tissue system	-	Dickey, Zemp 2011
miR-15b	2Gy	0.5, 168h	gamma	human 3D tissue system	-	Dickey, Zemp 2011
miR-15b	8Gy	1h	gamma	hFOB	+	Li, Ha 2012
miR-15b	2.5Gy	8, 24h	gamma	CRL2741	-	Weidhaas, Babar 2007
miR-15b	2.5Gy	2, 8, 24h	gamma	A549	-	Weidhaas, Babar 2007
miR-15b	8Gy	6h	gamma	HeLa	-	Hu, Tie 2013
miR-15b	1Gy	24h	Cs-137	B lymphoblasts IM9	-	Cha, Shin 2009
miR-15b-5p	1.3Gy	24h	^{177}Lu-octreotate	mouse renal cortical tissue	+	present study
miR-16	0.2Gy	0.5, 48h	gamma	human 3D tissue system	+	Dickey, Zemp 2011
miR-16	2Gy	0.5, 48, 168h	gamma	human 3D tissue system	+-	Dickey, Zemp 2011
miR-16	2 Gy	6h	6MV gamma	endoth cells	+	Wagner-Ecker, Schwager 2010
miR-16	20, 40Gy	24h	Cs-137	lung carcinoma cell A549	-	Shin, Cha 2009
miR-16	2.5Gy	2, 8, 24h	gamma	CRL2741	-	Weidhaas, Babar 2007
miR-16	2.5Gy	2, 24h	gamma	A549	-	Weidhaas, Babar 2007

miR-16	1.25Gy	4h	gamma	human blood (TBI)	+	Templin, Paul 2011
miR-16	1Gy	24h	Cs-137	B lymphoblasts IM9	-	Cha, Shin 2009
miR-16	8Gy	6h	gamma	HeLa	+	Hu, Tie 2013
miR-16	0.2Gy	4h	Cs-137	human PBL	+	Girardi, De Pitta 2012
miR-16-1	0.05Gy	8h	Cs-137	B lymphoblasts IM9	-	Cha, Seong 2009
miR-16-2-3p	2Gy	24h	Cs-137	human PBL	-	Girardi, De Pitta 2012
miR-17	0.2Gy	168h	gamma	human 3D tissue system	-	Dickey, Zemp 2011
miR-17	2Gy	168h	gamma	human 3D tissue system	-	Dickey, Zemp 2011
miR-17	1.25Gy	4h	gamma	human blood (TBI)	+	Templin, Paul 2011
miR-17	2Gy	24h	Cs-137	human PBL	-	Girardi, De Pitta 2012
miR-17-3p	1Gy	6h	90 kV x-ray	Male mouse cerebellum	+	Koturbash, Zemp
miR-17-3p	10 Gy	5, 15, 40h	gamma	PC3	-	Leung, Li 2014
miR-17-5p	6Gy	3h	Cs-137	proliferating kerotinocytes	-	Joly-Tonetti, Vinuelas
miR-17-5p	2.5Gy	2, 8, 24h	gamma	CRL2741	-	Weidhaas, Babar 2007
miR-17-5p	2.5Gy	2, 8h	gamma	A549	-	Weidhaas, Babar 2007
miR-17-5p	0.05Gy	8h	Cs-137	B lymphoblasts IM9	-	Cha, Seong 2009
miR-17-5p	1Gy	24h	Cs-137	B lymphoblasts IM9	-	Cha, Shin 2009
miR-17-5p	8Gy	6h	gamma	HeLa	+	Hu, Tie 2013
miR-17-5p	0.34Gy	24h	^{177}Lu-octreotate	mouse renal cortical tissue	+	present study
miR-18	0.05Gy	8h	Cs-137	B lymphoblasts IM9	-	Cha, Seong 2009
miR-18a	2 Gy	6h	6MV gamma	endoth cells	-	Wagner-Ecker, Schwager 2010
miR-18a	2 Gy	6h	250 keV x-rays	foreskin fibroblasts	-	Maes, An 2008
miR-18a	1Gy	24h	Cs-137	B lymphoblasts IM9	-	Cha, Shin 2009
miR-18b	2.5Gy	8h	gamma	CRL2741	+	Weidhaas, Babar 2007
miR-18b	2Gy	24h	Cs-137	human PBL	-	Girardi, De Pitta 2012
miR-19a	2.5Gy	2, 8, 24h	gamma	CRL2741	-	Weidhaas, Babar 2007
miR-19a	2.5Gy	2h	gamma	A549	-	Weidhaas, Babar 2007
miR-19a	1.25Gy	4h	gamma	human blood (TBI)	+	Templin, Paul 2011
miR-19a	8Gy	6h	gamma	HeLa	+	Hu, Tie 2013
miR-19a	0.05Gy	8h	Cs-137	B lymphoblasts IM9	-	Cha, Seong 2009
miR-19a-3p	6Gy	3h	Cs-137	differentiated kerotinocytes	+	Joly-Tonetti, Vinuelas
miR-19b	8Gy	1h	gamma	hFOB	-	Li, Ha 2012
miR-19b	2.5Gy	2, 8, 24h	gamma	CRL2741	-	Weidhaas, Babar 2007

miR-19b	2.5Gy	2, 8, 24h	gamma	A549	-	Weidhaas, Babar 2007
miR-19b	1Gy	24h	Cs-137	B lymphoblasts IM9	-	Cha, Shin 2009
miR-19b	8Gy	6h	gamma	HeLa	-	Hu, Tie 2013
miR-19b	2Gy	24h	Cs-137	human PBL	-	Girardi, De Pitta 2012
miR-20	0.05Gy	8h	Cs-137	B lymphoblasts IM9	-	Cha, Seong 2009
miR-20a	0.2, 2Gy	0.5, 48, 168h	gamma	human 3D tissue system	+-	Dickey, Zemp 2011
miR-20a	2 Gy	6h	6MV gamma	endoth cells	+	Wagner-Ecker, Schwager 2010
miR-20a	2.5Gy	8h	gamma	CRL2741	+	Weidhaas, Babar 2007
miR-20a	1.25Gy	4h	gamma	human blood (TBI)	+	Templin, Paul 2011
miR-20a	1Gy	24h	Cs-137	B lymphoblasts IM9	-	Cha, Shin 2009
miR-20a	2.5Gy	6h	90 kV x-ray	Male mouse spleen	-	Ilnytskyy, Zemp 2008
miR-20b	2.5Gy	2, 8, 24h	gamma	CRL2741	-	Weidhaas, Babar 2007
miR-20b	2.5Gy	2, 8, 24h	gamma	A549	-	Weidhaas, Babar 2007
miR-20b	1.25Gy	4h	gamma	human blood (TBI)	+	Templin, Paul 2011
miR-20b	8Gy	6h	gamma	HeLa	+	Hu, Tie 2013
miR-20b	1Gy	96h	90 kV x-ray	Female mouse hippocampus	+	Koturbash, Zemp
miR-20b-5p	0.34, 4.3 Gy	24h	^{177}Lu-octreotate	mouse renal cortical tissue	+	present study
miR-21	0.25, 0.5, 1, 3, 5, 10 Gy	1h	Co-60	NorHuFib	+	Simone, Soule 2009
miR-21	2Gy	168h	gamma	human 3D tissue system	+	Dickey, Zemp 2011
miR-21	2 Gy	6h	6MV gamma	endoth cells	+	Wagner-Ecker, Schwager 2010
miR-21	2.5Gy	2, 8, 24h	gamma	A549	+-	Weidhaas, Babar 2007
miR-21	1.25Gy	4h	gamma	human blood (TBI)	+	Templin, Paul 2011
miR-21	0.05Gy	8h	Cs-137	B lymphoblasts IM9	-	Cha, Seong 2009
miR-21	8Gy	6h	gamma	HeLa	+	Hu, Tie 2013
miR-21	1Gy	24h	Cs-137	B lymphoblasts IM9	-	Cha, Shin 2009
miR-21-3p	0.2, 2Gy	4h	Cs-137	human PBL	-	Girardi, De Pitta 2012
miR-21a-5p	13Gy	24h	^{177}Lu-octreotate	mouse renal cortical tissue	+	present study
miR-22	0.2, 2Gy	0.5, 168h	gamma	human 3D tissue system	+-	Dickey, Zemp 2011
miR-22	40Gy	24h	Cs-137	lung carcinoma cell A549	+	Shin, Cha 2009
miR-22	6Gy	24h	15 MeV photons	prostate cancer cells LNCaP	+	Li, Shi 2011
miR-22	2.5Gy	8, 24h	gamma	CRL2741	+	Weidhaas, Babar 2007
miR-22	2.5Gy	2, 8, 24h	gamma	A549	+	Weidhaas, Babar 2007
miR-22	18.8Gy	4h	6 MV photon	U87MG glioblastoma	+	Chen, Zhu 2009

miR-22-3p	4.3, 13Gy	24h	^{177}Lu-octreotate	mouse renal cortical tissue	+	present study
miR-22-3p	10 Gy	5, 15, 40h	gamma	PC3	+	Leung, Li 2014
miR-22-5p	1Gy	6h	90 kV x-ray	Female mouse frontal lobe	-	Koturbash, Zemp
miR-23a	0.2Gy	48, 168h	gamma	human 3D tissue system	-	Dickey, Zemp 2011
miR-23a	2Gy	48, 168h	gamma	human 3D tissue system	-	Dickey, Zemp 2011
miR-23a	2.5Gy	2, 8, 24h	gamma	CRL2741	-	Weidhaas, Babar 2007
miR-23a	2.5Gy	8h	gamma	A549	-	Weidhaas, Babar 2007
miR-23a	8Gy	6h	gamma	HeLa	+	Hu, Tie 2013
miR-23a	0.05Gy	8h	Cs-137	B lymphoblasts IM9	-	Cha, Seong 2009
miR-23a-5p	0.2Gy	4h	Cs-137	human PBL	+	Girardi, De Pitta 2012
miR-23b	0.2Gy	48, 168h	gamma	human 3D tissue system	-	Dickey, Zemp 2011
miR-23b	2Gy	48, 168h	gamma	human 3D tissue system	-	Dickey, Zemp 2011
miR-23b	2Gy	1h	gamma	CD34+	+	Li, Ha 2012
miR-23b	2.5Gy	2, 8, 24h	gamma	CRL2741	-	Weidhaas, Babar 2007
miR-23b	0.05Gy	8h	Cs-137	B lymphoblasts IM9	-	Cha, Seong 2009
miR-24	0.25, 0.5, 1, 3, 5, 10Gy	1h	Co-60	NorHuFib	-	Simone, Soule 2009
miR-24	0.2Gy	0.5, 168h	gamma	human 3D tissue system	+-	Dickey, Zemp 2011
miR-24	2Gy	0.5, 48, 168h	gamma	human 3D tissue system	+	Dickey, Zemp 2011
miR-24	6Gy	24h	15 MeV photons	prostate cancer cells LNCaP	+	Li, Shi 2011
miR-24	2.5Gy	2, 24h	gamma	CRL2741	-	Weidhaas, Babar 2007
miR-24	2.5Gy	2, 8, 24h	gamma	A549	-	Weidhaas, Babar 2007
miR-24	1.25Gy	4h	gamma	human blood (TBI)	+	Templin, Paul 2011
miR-24	8Gy	6h	gamma	HeLa	+	Hu, Tie 2013
miR-24	1Gy	24h	Cs-137	B lymphoblasts IM9	-	Cha, Shin 2009
miR-25	0.2Gy	0.5h	gamma	human 3D tissue system	-	Dickey, Zemp 2011
miR-25	2Gy	0.5, 48h	gamma	human 3D tissue system	+-	Dickey, Zemp 2011
miR-25	2.5Gy	2h	gamma	CRL2741	-	Weidhaas, Babar 2007
miR-25	8Gy	6h	gamma	HeLa	+	Hu, Tie 2013
miR-25	2.5Gy	2h	gamma	A549	-	Weidhaas, Babar 2007
miR-25-3p	10 Gy	5, 15, 40h	gamma	PC3	+	Leung, Li 2014
miR-26a	0.2Gy	0.5, 48h	gamma	human 3D tissue system	-	Dickey, Zemp 2011
miR-26a	2.5Gy	2h	gamma	A549	-	Weidhaas, Babar 2007
miR-26a-5p	6Gy	3h	Cs-137	proliferating kerotinocytes	-	Joly-Tonetti, Vinuelas

miR-26b	0.25, 0.5, 1, 3, 5, 10Gy	1h	Co-60	NorHuFib	+	Simone, Soule 2009
miR-26b	0.2, 2Gy	0.5, 48, 168h	gamma	human 3D tissue system	+-	Dickey, Zemp 2011
miR-26b	2.5Gy	24h	gamma	CRL2741	-	Weidhaas, Babar 2007
miR-26b	8Gy	6h	gamma	HeLa	-	Hu, Tie 2013
miR-26b	1.25Gy	4h	gamma	human blood (TBI)	+	Templin, Paul 2011
miR-26b-5p	4.3Gy	24h	^{177}Lu-octreotate	mouse renal cortical tissue	+	present study
miR-27a	0.2Gy	0.5, 48, 168h	gamma	human 3D tissue system	+-	Dickey, Zemp 2011
miR-27a	2Gy	0.5, 48, 168h	gamma	human 3D tissue system	+-	Dickey, Zemp 2011
miR-27a	2.5Gy	2, 8, 24h	gamma	CRL2741	-	Weidhaas, Babar 2007
miR-27a	2.5Gy	2, 8, 24h	gamma	A549	-	Weidhaas, Babar 2007
miR-27a	8Gy	6h	gamma	HeLa	+	Hu, Tie 2013
miR-27a	1.25Gy	4h	gamma	human blood (TBI)	+	Templin, Paul 2011
miR-27a	1Gy	96h	90 kV x-ray	Male mouse frontal lobe	-	Koturbash, Zemp
miR-27b	0.2Gy	168h	gamma	human 3D tissue system	-	Dickey, Zemp 2011
miR-27b	2Gy	0.5, 48, 168h	gamma	human 3D tissue system	+-	Dickey, Zemp 2011
miR-27b	8Gy	1h	gamma	hFOB	+	Li, Ha 2012
miR-27b	2.5Gy	2, 8, 24h	gamma	CRL2741	-	Weidhaas, Babar 2007
miR-27b	2.5Gy	8, 24h	gamma	A549	-	Weidhaas, Babar 2007
miR-27b	8Gy	6h	gamma	HeLa	-	Hu, Tie 2013
miR-27b	1Gy	96h	90 kV x-ray	Male mouse frontal lobe	-	Koturbash, Zemp
miR-28	2.5Gy	2, 24h	gamma	A549	+	Weidhaas, Babar 2007
miR-28-3p	6Gy	3h	Cs-137	differentiated kerotinocytes	+	Joly-Tonetti, Vinuelas
miR-29a	0.2Gy	0.5, 168h	gamma	human 3D tissue system	+-	Dickey, Zemp 2011
miR-29a	2Gy	0.5, 168h	gamma	human 3D tissue system	+-	Dickey, Zemp 2011
miR-29a	2.5Gy	2, 8, 24h	gamma	CRL2741	-	Weidhaas, Babar 2007
miR-29a	2.5Gy	2, 8, 24h	gamma	A549	-	Weidhaas, Babar 2007
miR-29a	1.25Gy	4h	gamma	human blood (TBI)	+	Templin, Paul 2011
miR-29a	1Gy	24h	Cs-137	B lymphoblasts IM9	-	Cha, Shin 2009
miR-29a	8Gy	6h	gamma	HeLa	+	Hu, Tie 2013
miR-29a	1Gy	6, 96h	90 kV x-ray	Female mouse frontal lobe	-	Koturbash, Zemp
miR-29b	6Gy	24h	15 MeV photons	prostate cancer cells LNCaP	+	Li, Shi 2011
miR-29b	2.5Gy	2, 8, 24h	gamma	CRL2741	-	Weidhaas, Babar 2007
miR-29b	2.5Gy	2, 8, 24h	gamma	A549	-	Weidhaas, Babar 2007

miR-29b	2.5Gy	6h	90 kV x-ray	Female mouse spleen	-	Ilnytskyy, Zemp 2008
miR-29b-1	8Gy	1h	gamma	hFOB	+	Li, Ha 2012
miR-29b-1-5p	2Gy	24h	Cs-137	human PBL	-	Girardi, De Pitta 2012
miR-29c	0.2Gy	48h	gamma	human 3D tissue system	+	Dickey, Zemp 2011
miR-29c	2Gy	0.5, 168h	gamma	human 3D tissue system	+-	Dickey, Zemp 2011
miR-29c	2 Gy	6h	6MV gamma	endoth cells	+	Wagner-Ecker, Schwager 2010
miR-29c	8Gy	1h	gamma	hFOB	+	Li, Ha 2012
miR-29c	2.5Gy	24h	gamma	CRL2741	+	Weidhaas, Babar 2007
miR-29c	2.5Gy	2, 8h	gamma	A549	+	Weidhaas, Babar 2007
miR-29c	1.25Gy	4h	gamma	human blood (TBI)	+	Templin, Paul 2011
miR-29c	8Gy	6h	gamma	HeLa	+	Hu, Tie 2013
miR-29c	10Gy	24h	Cs-137	B lymphoblasts IM9	+	Cha, Shin 2009
miR-29c-5p	1Gy	6, 96h	90 kV x-ray	Female mouse frontal lobe	-	Koturbash, Zemp
miR-29c-5p	1Gy	96h	90 kV x-ray	Male mouse frontal lobe	-	Koturbash, Zemp
miR-30a	0.2, 2Gy	0.5, 48, 168h	gamma	human 3D tissue system	+-	Dickey, Zemp 2011
miR-30a-5p	6Gy	24h	15 MeV photons	prostate cancer cells LNCaP	+	Li, Shi 2011
miR-30a-5p	2.5Gy	2, 8, 24h	gamma	CRL2741	-	Weidhaas, Babar 2007
miR-30a-5p	2.5Gy	2, 8, 24h	gamma	A549	-	Weidhaas, Babar 2007
miR-30a-5p	10 Gy	5, 15, 40h	gamma	PC3	+	Leung, Li 2014
miR-30b	0.2, 2Gy	0.5, 168h	gamma	human 3D tissue system	+-	Dickey, Zemp 2011
miR-30b	40Gy	24h	Cs-137	lung carcinoma cell A549	-	Shin, Cha 2009
miR-30b	2Gy	1h	gamma	CD34+	+	Li, Ha 2012
miR-30b	2.5Gy	2, 8, 24h	gamma	CRL2741	-	Weidhaas, Babar 2007
miR-30b	2.5Gy	2, 8, 24h	gamma	A549	-	Weidhaas, Babar 2007
miR-30b-5p	13Gy	24h	^{177}Lu-octreotate	mouse renal cortical tissue	+	present study
miR-30c	0.2Gy	0.5, 48, 168h	gamma	human 3D tissue system	+	Dickey, Zemp 2011
miR-30c	40Gy	24h	Cs-137	lung carcinoma cell A549	+	Shin, Cha 2009
miR-30c	2Gy	1h	gamma	CD34+	+	Li, Ha 2012
miR-30c	8Gy	1h	gamma	hFOB	-	Li, Ha 2012
miR-30c	2.5Gy	2, 8, 24h	gamma	CRL2741	-	Weidhaas, Babar 2007
miR-30c	2.5Gy	2, 8, 24h	gamma	A549	-	Weidhaas, Babar 2007
miR-30c-5p	4.3, 13Gy	24h	^{177}Lu-octreotate	mouse renal cortical tissue	+	present study
miR-30d	0.2, 2Gy	48, 168h	gamma	human 3D tissue system	+	Dickey, Zemp 2011

miR-30d	2Gy	1h	gamma	CD34+	+	Li, Ha 2012
miR-30d	2.5Gy	2, 24h	gamma	A549	+	Weidhaas,
miR-30d	2Gy	24h	Cs-137	human PBL	-	Girardi, De Pitta 2012
miR-30d-3p	10 Gy	5, 15, 40h	gamma	PC3	-	Leung, Li 2014
miR-30e	8Gy	1h	gamma	hFOB	+	Li, Ha 2012
miR-30e-3p	2.5Gy	2h	gamma	A549	+	Weidhaas, Babar 2007
miR-30e-3p	2Gy	24h	Cs-137	human PBL	-	Girardi, De Pitta 2012
miR-30e-5p	2.5Gy	24h	gamma	CRL2741	+	Weidhaas, Babar 2007
miR-30e-5p	2.5Gy	2h	gamma	A549	+	Weidhaas, Babar 2007
miR-30e-5p	1.3, 13Gy	24h	^{177}Lu-octreotate	mouse renal cortical tissue	+	present study
miR-31	0.2Gy	0.5, 48, 168h	gamma	human 3D tissue system	+-	Dickey, Zemp 2011
miR-31	2Gy	0.5, 48h	gamma	human 3D tissue system	+	Dickey, Zemp 2011
miR-31	2.5Gy	2, 8, 24h	gamma	CRL2741	-	Weidhaas, Babar 2007
miR-31	8Gy	6h	gamma	HeLa	-	Hu, Tie 2013
miR-31	2.5Gy	2, 8, 24h	gamma	A549	-	Weidhaas, Babar 2007
miR-31-5p	6Gy	3h	Cs-137	proliferating kerotinocytes	-	Joly-Tonetti, Vinuelas
miR-34a	40Gy	24h	Cs-137	lung carcinoma cell A549	+	Shin, Cha 2009
miR-34a	1Gy	24h	Cs-137	Normal thyroid cells	-	Nikiforova, Gandhi 2011
miR-34a	10Gy	4h	Cs-137	Normal thyroid cells	+	Nikiforova, Gandhi 2011
miR-34a	2.5Gy	8, 24h	gamma	A549	+	Weidhaas, Babar 2007
miR-34a	10Gy	24h	Cs-137	B lymphoblasts IM9	+	Cha, Shin 2009
miR-34a	2.5Gy	6h	90 kV x-ray	Male mouse spleen	+	Ilnytskyy, Zemp 2008
miR-34a	2.5Gy	6h	90 kV x-ray	Female mouse spleen	+	Ilnytskyy, Zemp 2008
miR-34a	0.2Gy	4h	Cs-137	human PBL	-	Girardi, De Pitta 2012
miR-34a	0.2, 2Gy	24h	Cs-137	human PBL	+	Girardi, De Pitta 2012
miR-34a	1Gy	6h	90 kV x-ray	Female mouse frontal lobe	-	Koturbash, Zemp
miR-34b	40Gy	24h	Cs-137	lung carcinoma cell A549	+	Shin, Cha 2009
miR-34b	1Gy	24h	Cs-137	Normal thyroid cells	+	Nikiforova, Gandhi 2011
miR-34b	10Gy	4, 24h	Cs-137	Normal thyroid cells	+	Nikiforova, Gandhi 2011
miR-34b	10Gy	24h	Cs-137	B lymphoblasts IM9	+	Cha, Shin 2009
miR-34b-5p	0.2, 2Gy	4h	Cs-137	human PBL	-	Girardi, De Pitta 2012
miR-34b-5p	0.2, 2Gy	24h	Cs-137	human PBL	+	Girardi, De Pitta 2012
miR-34c	1.0 Gy	6 h	p	mouse blood	-	Templin, Young 2012

miR-34c	6Gy	4h	6 MV photons	LNCaP prostate cancer cell	+	Josson, Sung 2008
miR-34c	6Gy	4h	6 MV photons	C4-2 prostate cancer cell	+	Josson, Sung 2008
miR-34c	1Gy	96h	90 kV x-ray	Female mouse hippocampus	+	Koturbash, Zemp
miR-34c	1Gy	6h	90 kV x-ray	Male mouse hippocampus	-	Koturbash, Zemp
miR-34c-3p	0.2Gy	0.5, 48, 168h	gamma	human 3D tissue system	+-	Dickey, Zemp 2011
miR-34c-3p	2Gy	0.5, 48, 168h	gamma	human 3D tissue system	+-	Dickey, Zemp 2011
miR-34c-5p	0.2Gy	0.5h	gamma	human 3D tissue system	+	Dickey, Zemp 2011
miR-92	2.5Gy	8h	gamma	CRL2741	-	Weidhaas, Babar 2007
miR-92	8Gy	6h	gamma	HeLa	+	Hu, Tie 2013
miR-92	1Gy	96h	90 kV x-ray	Female mouse frontal lobe	+	Koturbash, Zemp
miR-92a	2Gy	1h	gamma	CD34+	+	Li, Ha 2012
miR-92a-3p	0.34, 1.3, 4.3, 13Gy	24h	^{177}Lu-octreotate	mouse renal cortical tissue	+	present study
miR-92a-5p	10 Gy	5, 15, 40h	gamma	PC3	-	Leung, Li 2014
miR-92b	2 Gy	0.5h	250 keV x-rays	foreskin fibroblasts	-	Maes, An 2008
miR-92b	2Gy	1h	gamma	CD34+	+	Li, Ha 2012
miR-92b	2.5Gy	8h	gamma	CRL2741	-	Weidhaas, Babar 2007
miR-93	1Gy	4, 24h	Cs-137	Normal thyroid cells	-	Nikiforova, Gandhi 2011
miR-93	10Gy	4, 24h	Cs-137	Normal thyroid cells	-	Nikiforova, Gandhi 2011
miR-93	1.25Gy	4h	gamma	human blood (TBI)	+	Templin, Paul 2011
miR-93	1Gy	24h	Cs-137	B lymphoblasts IM9	-	Cha, Shin 2009
miR-93	2.5Gy	6h	90 kV x-ray	Male mouse spleen	-	Ilnytskyy, Zemp 2008
miR-93	1Gy	96h	90 kV x-ray	Male mouse frontal lobe	-	Koturbash, Zemp
miR-95	2Gy	24h	Cs-137	human PBL	-	Girardi, De Pitta 2012
miR-96	1Gy	4h	Cs-137	Normal thyroid cells	+	Nikiforova, Gandhi 2011
miR-96	10Gy	4h	Cs-137	Normal thyroid cells	+	Nikiforova, Gandhi 2011
miR-96	1Gy	6h	90 kV x-ray	Female mouse frontal lobe	+	Koturbash, Zemp
miR-98	2.5Gy	24h	gamma	CRL2741	-	Weidhaas, Babar 2007
miR-98	8Gy	6h	gamma	HeLa	-	Hu, Tie 2013
miR-98	2.5Gy	2h	gamma	A549	+	Weidhaas, Babar 2007
miR-99a	1.0 Gy	6 h	p	mouse blood	-	Templin, Young 2012
miR-99a	2.5Gy	8h	gamma	CRL2741	+	Weidhaas, Babar 2007
miR-99a	2.5Gy	2, 8, 24h	gamma	A549	+	Weidhaas, Babar 2007
miR-99a	8Gy	6h	gamma	HeLa	+	Hu, Tie 2013

miR-99a	2Gy	24h	Cs-137	human PBL		-	Girardi, De Pitta 2012
miR-99b	8Gy	6h	gamma	HeLa		+	Hu, Tie 2013
miR-99b	10Gy	8h	Cs-137	B lymphoblasts IM9		+	Cha, Seong 2009
miR-99b	2 Gy	4h	proton	Mouse brain	+		Khan, Tariq 2013
miR-99b	2 Gy	4h	proton	Mouse testis		+	Khan, Tariq 2013
miR-99b-5p	6Gy	3h	Cs-137	proliferating kerotinocytes		-	Joly-Tonetti, Vinuelas
miR-99b-5p	0.34Gy	24h	^{177}Lu-octreotate	mouse renal cortical tissue		+	present study
miR-100	0.25, 0.5, 1, 3, 5, 10Gy	1h	Co-60	NorHuFib		-	Simone, Soule 2009
miR-100	1.0 Gy	6 h	p	mouse blood		-	Templin, Young 2012
miR-100	6Gy	4h	6 MV photons	LNCaP prostate cancer cell		-	Josson, Sung 2008
miR-100	8Gy	1h	gamma	hFOB		-	Li, Ha 2012
miR-100	2.5Gy	8h	gamma	CRL2741		+	Weidhaas, Babar 2007
miR-100	2.5Gy	24h	gamma	A549		+	Weidhaas, Babar 2007
miR-100	8Gy	6h	gamma	HeLa		+	Hu, Tie 2013
miR-100	2Gy	24h	Cs-137	human PBL		-	Girardi, De Pitta 2012
miR-100	2 Gy	4h	proton	Mouse brain	+		Khan, Tariq 2013
miR-101	2.5Gy	8, 24h	gamma	CRL2741		+	Weidhaas, Babar 2007
miR-101	2.5Gy	2, 24h	gamma	A549		+	Weidhaas, Babar 2007
miR-101	8Gy	6h	gamma	HeLa		+	Hu, Tie 2013
miR-101	1.25Gy	4h	gamma	human blood (TBI)		+	Templin, Paul 2011
miR-101-5p	2Gy	24h	Cs-137	human PBL		-	Girardi, De Pitta 2012
miR-101a	2.5Gy	6h	90 kV x-ray	Male mouse thymus		+	Ilnytskyy, Zemp 2008
miR-101b	2.5Gy	6h	90 kV x-ray	Male mouse spleen		-	Ilnytskyy, Zemp 2008
miR-101b	1Gy	6, 96h	90 kV x-ray	Female mouse frontal lobe		-	Koturbash, Zemp
miR-101b	1Gy	96h	90 kV x-ray	Male mouse frontal lobe		-	Koturbash, Zemp
miR-103	0.2, 2Gy	0.5, 168h	gamma	human 3D tissue system		+	Dickey, Zemp 2011
miR-103	2Gy	1h	gamma	CD34+		-	Li, Ha 2012
miR-103	1.25Gy	4h	gamma	human blood (TBI)		+	Templin, Paul 2011
miR-103	1Gy	24h	Cs-137	B lymphoblasts IM9		-	Cha, Shin 2009
miR-106a	20, 40	24h	Cs-137	lung carcinoma cell A549		-	Shin, Cha 2009
miR-106a	2.5Gy	2, 8, 24h	gamma	CRL2741		-	Weidhaas, Babar 2007
miR-106a	2.5Gy	2, 8h	gamma	A549		-	Weidhaas, Babar 2007
miR-106a	1.25Gy	4h	gamma	human blood (TBI)		+	Templin, Paul 2011

miR-106a	0.05Gy	8h	Cs-137	B lymphoblasts IM9	-	Cha, Seong 2009
miR-106a	1Gy	24h	Cs-137	B lymphoblasts IM9	-	Cha, Shin 2009
miR-106a	8Gy	6h	gamma	HeLa	-	Hu, Tie 2013
miR-106a-5p	0.34, 1.3, 13Gy	24h	^{177}Lu-octreotate	mouse renal cortical tissue	+	present study
miR-106b	6Gy	24h	15 MeV photons	prostate cancer cells LNCaP	-	Li, Shi 2011
miR-106b	2.5Gy	8h	gamma	CRL2741	+	Weidhaas, Babar 2007
miR-106b	2.5Gy	24h	gamma	A549	+	Weidhaas, Babar 2007
miR-106b	1.25Gy	4h	gamma	human blood (TBI)	+	Templin, Paul 2011
miR-106b	0.05, 1Gy	8, 24h	Cs-137	B lymphoblasts IM9	-	Cha, Seong 2009
miR-106b	1Gy	96h	90 kV x-ray	Male mouse cerebellum	+	Koturbash, Zemp
miR-106b	1Gy	96h	90 kV x-ray	Male mouse frontal lobe	-	Koturbash, Zemp
miR-106b-5p	4.3, 13Gy	24h	^{177}Lu-octreotate	mouse renal cortical tissue	+	present study
miR-107	0.2, 2Gy	0.5, 48, 168h	gamma	human 3D tissue system	+-	Dickey, Zemp 2011
miR-107	6Gy	4h	6 MV photons	LNCaP prostate cancer cell	-	Josson, Sung 2008
miR-107	1Gy	24h	Cs-137	B lymphoblasts IM9	-	Cha, Shin 2009
miR-107	18.8Gy	4h	6 MV photon	U87MG glioblastoma	-	Chen, Zhu 2009
miR-107-3p	0.34, 1.3, 4.3, 13Gy	24h	^{177}Lu-octreotate	mouse renal cortical tissue	+	present study
miR-122a	6Gy	4h	6 MV photons	LNCaP prostate cancer cell	-	Josson, Sung 2008
miR-122a	1Gy	4, 24h	Cs-137	Normal thyroid cells	+	Nikiforova, Gandhi 2011
miR-122a	10Gy	4, 24h	Cs-137	Normal thyroid cells	+	Nikiforova, Gandhi 2011
miR-123a	1Gy	6h	90 kV x-ray	Male mouse hippocampus	+	Koturbash, Zemp
miR-125a	2 Gy	6h	6MV gamma	endoth cells	-	Wagner-Ecker, Schwager 2010
miR-125a	2.5Gy	8, 24h	gamma	CRL2741	-	Weidhaas, Babar 2007
miR-125a	2.5Gy	2, 8, 24h	gamma	A549	-	Weidhaas, Babar 2007
miR-125a	1Gy	6h	90 kV x-ray	Female mouse hippocampus	-	Koturbash, Zemp
miR-125a-3p	5Gy	24h	Cs-137	Mouse blood	+	Templin, Amundson 2011
miR-125a-3p	0.1Gy	6h	Fe-56	Mouse blood	-	Templin, Amundson 2011
miR-125a-3p	10 Gy	5, 15, 40h	gamma	PC3	-	Leung, Li 2014
miR-125a-5p	0.2Gy	0.5, 48h	gamma	human 3D tissue system	+-	Dickey, Zemp 2011
miR-125a-5p	2Gy	0.5h	gamma	human 3D tissue system	-	Dickey, Zemp 2011
miR-125a-5p	0.34, 1.3Gy	24h	^{177}Lu-octreotate	mouse renal cortical tissue	+	present study
miR-125b	0.25, 0.5, 1, 3, 5, 10Gy	1h	Co-60	NorHuFib	-	Simone, Soule 2009

miR-125b	0.2Gy	48, 168h	gamma	human 3D tissue system	+	Dickey, Zemp 2011
miR-125b	8Gy	1h	gamma	hFOB	-	Li, Ha 2012
miR-125b	2.5Gy	2h	gamma	CRL2741	-	Weidhaas, Babar 2007
miR-125b	2.5Gy	24h	gamma	A549	-	Weidhaas, Babar 2007
miR-125b-5p	1.0 Gy	6 h	p	mouse blood	-	Templin, Young 2012
miR-125b-5p	6Gy	3h	Cs-137	differentiated kerotinocytes	-	Joly-Tonetti, Vinuelas
miR-125b-5p	0.34Gy	24h	^{177}Lu-octreotate	mouse renal cortical tissue	+	present study
miR-126	2.5Gy	2, 24h	gamma	A549	+	Weidhaas, Babar 2007
miR-126	1.25Gy	4h	gamma	human blood (TBI)	+	Templin, Paul 2011
miR-126-5p	2Gy	4h	Cs-137	human PBL	-	Girardi, De Pitta 2012
miR-127	1.0 Gy	6 h	p	mouse blood	-	Templin, Young 2012
miR-127	2 Gy	6h	6MV gamma	endoth cells	-	Wagner-Ecker, Schwager 2010
miR-127	2 Gy	4h	proton	Mouse liver	+	Khan, Tariq 2013
miR-127-3p	20, 40Gy	24h	Cs-137	lung carcinoma cell A549	+	Shin, Cha 2009
miR-127-3p	8Gy	1h	gamma	hFOB	-	Li, Ha 2012
miR-129-5p	1Gy	6h	90 kV x-ray	Female mouse cerebellum	+	Koturbash, Zemp
miR-130a	2.5Gy	8h	gamma	CRL2741	+	Weidhaas, Babar 2007
miR-130a	1.25Gy	4h	gamma	human blood (TBI)	+	Templin, Paul 2011
miR-130a	1Gy	6h	90 kV x-ray	Female mouse cerebellum	+	Koturbash, Zemp
miR-130b	2Gy	1h	gamma	CD34+	-	Li, Ha 2012
miR-130b	1Gy	96h	90 kV x-ray	Male mouse hippocampus	-	Koturbash, Zemp
miR-132	2.5Gy	24h	gamma	CRL2741	+	Weidhaas, Babar 2007
miR-133a	1Gy	96h	90 kV x-ray	Female mouse hippocampus	-	Koturbash, Zemp
miR-133b	6Gy	4h	6 MV photons	LNCaP prostate cancer cell	-	Josson, Sung 2008
miR-133b	6Gy	4h	6 MV photons	C4-2 prostate cancer cell	-	Josson, Sung 2008
miR-134	1, 10Gy	24h	Cs-137	B lymphoblasts IM9	+	Cha, Shin 2009
miR-135a	1.5, 5Gy	6h	Cs-137	Mouse blood	+	Templin, Amundson 2011
miR-135a	1Gy	96h	90 kV x-ray	Female mouse hippocampus	+	Koturbash, Zemp
miR-135a-3p	0.2, 2Gy	24h	Cs-137	human PBL	+	Girardi, De Pitta 2012
miR-135b	6Gy	4h	6 MV photons	C4-2 prostate cancer cell	-	Josson, Sung 2008
miR-135b	5Gy	6h	Cs-137	Mouse blood	+	Templin, Amundson 2011
miR-137	0.1 Gy	24h	250 keV x-rays	foreskin fibroblasts	-	Maes, An 2008
miR-138	2.5Gy	2h	gamma	A549	+	Weidhaas, Babar 2007

miR-138	2 Gy	4h	proton	Mouse testis	-	Khan, Tariq 2013
miR-139-3p	20 Gy	24h	Cs-137	lung carcinoma cell A549	-	Shin, Cha 2009
miR-139-5p	5Gy	6h	Cs-137	Mouse blood	+	Templin, Amundson 2011
miR-140-3p	1Gy	6h	90 kV x-ray	Female mouse frontal lobe	-	Koturbash, Zemp
miR-140-3p	0.34Gy	24h	^{177}Lu-octreotate	mouse renal cortical tissue	+	present study
miR-140-5p	1.25Gy	4h	gamma	human blood (TBI)	+	Templin, Paul 2011
miR-141	0.2Gy	0.5, 48h	gamma	human 3D tissue system	+	Dickey, Zemp 2011
miR-141	2Gy	0.5, 48h	gamma	human 3D tissue system	+	Dickey, Zemp 2011
miR-141	6Gy	24h	15 MeV photons	prostate cancer cells LNCaP	+	Li, Shi 2011
miR-141	2.5Gy	24h	gamma	CRL2741	+	Weidhaas, Babar 2007
miR-141	2Gy	24h	Cs-137	human PBL	-	Girardi, De Pitta 2012
miR-141	1Gy	6h	90 kV x-ray	Female mouse frontal lobe	+	Koturbash, Zemp
miR-142-3p	1.25Gy	4h	gamma	human blood (TBI)	+	Templin, Paul 2011
miR-142-3p	1Gy	24h	Cs-137	B lymphoblasts IM9	-	Cha, Shin 2009
miR-142-5p	1.25Gy	4h	gamma	human blood (TBI)	+	Templin, Paul 2011
miR-142-5p	1Gy	24h	Cs-137	B lymphoblasts IM9	-	Cha, Shin 2009
miR-143	1.0 Gy	6 h	p	mouse blood	-	Templin, Young 2012
miR-143	6Gy	4h	6 MV photons	C4-2 prostate cancer cell	-	Josson, Sung 2008
miR-143	1.25Gy	4h	gamma	human blood (TBI)	+	Templin, Paul 2011
miR-143	1Gy	6h	90 kV x-ray	Female mouse frontal lobe	-	Koturbash, Zemp
miR-145	6Gy	4h	6 MV photons	LNCaP prostate cancer cell	-	Josson, Sung 2008
miR-145	1.25Gy	4h	gamma	human blood (TBI)	+	Templin, Paul 2011
miR-145	0.2Gy	4h	Cs-137	human PBL	+	Girardi, De Pitta 2012
miR-146	1Gy	6h	90 kV x-ray	Female mouse frontal lobe	-	Koturbash, Zemp
miR-146a	1Gy	4, 24h	Cs-137	Normal thyroid cells	+-	Nikiforova, Gandhi 2011
miR-146a	10Gy	24h	Cs-137	Normal thyroid cells	-	Nikiforova, Gandhi 2011
miR-146a	5Gy	24h	Cs-137	Mouse blood	-	Templin, Amundson 2011
miR-146a	1Gy	24h	Cs-137	B lymphoblasts IM9	-	Cha, Shin 2009
miR-146a	2Gy	4h	Cs-137	human PBL	-	Girardi, De Pitta 2012
miR-146b	2 Gy	4h	proton	Mouse testis	+	Khan, Tariq 2013
miR-147	1.5, 5Gy	6h	Cs-137	Mouse blood	+	Templin, Amundson 2011
miR-148a	2.5Gy	2, 8, 24h	gamma	CRL2741	-	Weidhaas, Babar 2007
miR-148a	1.25Gy	4h	gamma	human blood (TBI)	+	Templin, Paul 2011

miR-148b	2 Gy	6h	6MV gamma	endoth cells	-	Wagner-Ecker, Schwager 2010
miR-148b	2.5Gy	24h	gamma	CRL2741	+	Weidhaas, Babar 2007
miR-148b	2.5Gy	2, 24h	gamma	A549	+	Weidhaas, Babar 2007
miR-148b	1.25Gy	4h	gamma	human blood (TBI)	+	Templin, Paul 2011
miR-148b	8Gy	6h	gamma	HeLa	+	Hu, Tie 2013
miR-148b	1Gy	6h	90 kV x-ray	Female mouse cerebellum	+	Koturbash, Zemp
miR-149	0.2Gy	48, 168h	gamma	human 3D tissue system	+-	Dickey, Zemp 2011
miR-149	2Gy	0.5, 48, 168h	gamma	human 3D tissue system	+-	Dickey, Zemp 2011
miR-149	2Gy	1h	gamma	CD34+	-	Li, Ha 2012
miR-149	1Gy	96h	90 kV x-ray	Male mouse frontal lobe	-	Koturbash, Zemp
miR-150	1.5Gy	6h	Cs-137	Mouse blood	-	Templin, Amundson 2011
miR-150	5Gy	24h	Cs-137	Mouse blood	-	Templin, Amundson 2011
miR-150	0.5Gy	24h	Fe-56	Mouse blood	-	Templin, Amundson 2011
miR-150	2 Gy	4h	proton	Mouse liver	+	Khan, Tariq 2013
miR-150-3p	0.2Gy	24h	Cs-137	human PBL	+	Girardi, De Pitta 2012
miR-151	1Gy	96h	90 kV x-ray	Female mouse hippocampus	-	Koturbash, Zemp
miR-151-3p	8Gy	1h	gamma	hFOB	-	Li, Ha 2012
miR-151-3p	5Gy	24h	Cs-137	Mouse blood	+	Templin, Amundson 2011
miR-151-3p	2Gy	24h	Cs-137	human PBL	-	Girardi, De Pitta 2012
miR-151-5p	0.2, 2Gy	48, 168h	gamma	human 3D tissue system	+	Dickey, Zemp 2011
miR-151-5p	0.34, 1.3Gy	24h	^{177}Lu-octreotate	mouse renal cortical tissue	+	present study
miR-152	2.5Gy	2h	gamma	A549	-	Weidhaas, Babar 2007
miR-152	0.2, 2Gy	24h	Cs-137	human PBL	-	Girardi, De Pitta 2012
miR-153	1Gy	96h	90 kV x-ray	Female mouse frontal lobe	-	Koturbash, Zemp
miR-154	6Gy	4h	6 MV photons	C4-2 prostate cancer cell	+	Josson, Sung 2008
miR-154	1Gy	96h	90 kV x-ray	Female mouse hippocampus	-	Koturbash, Zemp
miR-154	1Gy	6h	90 kV x-ray	Female mouse frontal lobe	-	Koturbash, Zemp
miR-155	2.5Gy	2, 8, 24h	gamma	CRL2741	-	Weidhaas, Babar 2007
miR-155	0.05Gy	8h	Cs-137	B lymphoblasts IM9	-	Cha, Seong 2009
miR-155	1Gy	24h	Cs-137	B lymphoblasts IM9	-	Cha, Shin 2009
miR-155	8Gy	6h	gamma	HeLa	-	Hu, Tie 2013
miR-155	1Gy	96h	90 kV x-ray	Female mouse hippocampus	+	Koturbash, Zemp
miR-181a	0.2, 2Gy	0.5, 168h	gamma	human 3D tissue system	+-	Dickey, Zemp 2011

miR-181a	2.5Gy	2hh	gamma	A549	+	Weidhaas, Babar 2007
miR-181a	18.8Gy	4h	6 MV photon	U87MG glioblastoma	-	Chen, Zhu 2009
miR-181a	2Gy	24h	Cs-137	human PBL	-	Girardi, De Pitta 2012
miR-181a-5p	6Gy	3h	Cs-137	differentiated kerotinocytes	+	Joly-Tonetti, Vinuelas
miR-181a-5p	0.34, 13Gy	24h	^{177}Lu-octreotate	mouse renal cortical tissue	+	present study
miR-181a-2-3p	0.2, 2Gy	24h	Cs-137	human PBL	-	Girardi, De Pitta 2012
miR-181b	2 Gy	6h	250 keV x-rays	foreskin fibroblasts	-	Maes, An 2008
miR-181b	2.5Gy	6h	90 kV x-ray	Female mouse spleen	-	Ilnytskyy, Zemp 2008
miR-181b	0.2Gy	4h	Cs-137	human PBL	+	Girardi, De Pitta 2012
miR-181b	8Gy	6h	gamma	HeLa	+	Hu, Tie 2013
miR-181b	1Gy	6h	90 kV x-ray	Male mouse hippocampus	-	Koturbash, Zemp
miR-181c	1, 10Gy	4, 24h	Cs-137	Normal thyroid cells	-	Nikiforova, Gandhi 2011
miR-181d	2.5Gy	2, 8h	gamma	CRL2741	-	Weidhaas, Babar 2007
miR-181d	2.5Gy	2, 8, 24h	gamma	A549	-	Weidhaas, Babar 2007
miR-181d	8Gy	6h	gamma	HeLa	-	Hu, Tie 2013
miR-181d	1Gy	96h	90 kV x-ray	Female mouse cerebellum	+	Koturbash, Zemp
miR-182	0.2Gy	0.5h	gamma	human 3D tissue system	-	Dickey, Zemp 2011
miR-182	2Gy	0.5, 168h	gamma	human 3D tissue system	+-	Dickey, Zemp 2011
miR-182	2.5Gy	8, 24h	gamma	CRL2741	-	Weidhaas, Babar 2007
miR-182	8Gy	6h	gamma	HeLa	+	Hu, Tie 2013
miR-182	2.5Gy	2, 8, 24h	gamma	A549	-	Weidhaas, Babar 2007
miR-182-3p	2.5Gy	8, 24h	gamma	CRL2741	-	Weidhaas, Babar 2007
miR-183	40Gy	24h	Cs-137	lung carcinoma cell A549	+	Shin, Cha 2009
miR-183	2.5Gy	24h	gamma	CRL2741	-	Weidhaas, Babar 2007
miR-183	2.5Gy	2h	gamma	A549	-	Weidhaas, Babar 2007
miR-184	2.5Gy	8h	gamma	A549	-	Weidhaas, Babar 2007
miR-185	2.5Gy	2h	gamma	A549	-	Weidhaas, Babar 2007
miR-185	1.25Gy	4h	gamma	human blood (TBI)	+	Templin, Paul 2011
miR-186	1Gy	4, 24h	Cs-137	Normal thyroid cells	-	Nikiforova, Gandhi 2011
miR-186	10Gy	4, 24h	Cs-137	Normal thyroid cells	-	Nikiforova, Gandhi 2011
miR-186	8Gy	6h	gamma	HeLa	-	Hu, Tie 2013
miR-186	1Gy	96h	90 kV x-ray	Female mouse hippocampus	+	Koturbash, Zemp
miR-186-5p	6Gy	3h	Cs-137	differentiated kerotinocytes	+	Joly-Tonetti, Vinuelas

miR-187	6Gy	4h	6 MV photons	LNCaP prostate cancer cell	-	Josson, Sung 2008
miR-187	10Gy	8h	Cs-137	B lymphoblasts IM9	+	Cha, Seong 2009
miR-187	1Gy	6h	90 kV x-ray	Female mouse cerebellum	+	Koturbash, Zemp
miR-188	1Gy	4, 24h	Cs-137	Normal thyroid cells	+	Nikiforova, Gandhi 2011
miR-188	10Gy	4, 24h	Cs-137	Normal thyroid cells	+	Nikiforova, Gandhi 2011
miR-188-5p	2 Gy	6h	250 keV x-rays	foreskin fibroblasts	-	Maes, An 2008
miR-188-5p	2Gy	4h	Cs-137	human PBL	-	Girardi, De Pitta 2012
miR-188-5p	0.2, 2Gy	24h	Cs-137	human PBL	+	Girardi, De Pitta 2012
miR-189	2 Gy	6h	6MV gamma	endoth cells	-	Wagner-Ecker, Schwager 2010
miR-190	1.25Gy	4h	gamma	human blood (TBI)	+	Templin, Paul 2011
miR-191	2Gy	48, 168h	gamma	human 3D tissue system	+-	Dickey, Zemp 2011
miR-191	6Gy	24h	15 MeV photons	prostate cancer cells LNCaP	+	Li, Shi 2011
miR-191	2.5Gy	2, 24h	gamma	CRL2741	-	Weidhaas, Babar 2007
miR-191	2.5Gy	2, 24h	gamma	A549	-	Weidhaas, Babar 2007
miR-191	18.8Gy	4h	6 MV photon	U87MG glioblastoma	+	Chen, Zhu 2009
miR-191-3p	2.5Gy	2, 24h	gamma	CRL2741	-	Weidhaas, Babar 2007
miR-191-5p	6Gy	3h	Cs-137	proliferating kerotinocytes	-	Joly-Tonetti, Vinuelas
miR-192	40Gy	24h	Cs-137	lung carcinoma cell A549	+	Shin, Cha 2009
miR-192	2 Gy	0.5h	250 keV x-rays	foreskin fibroblasts	-	Maes, An 2008
miR-192	2.5Gy	6h	90 kV x-ray	Male mouse spleen	-	Ilnytskyy, Zemp 2008
miR-192	1Gy	6h	90 kV x-ray	Female mouse frontal lobe	-	Koturbash, Zemp
miR-193a	1Gy	4, 24h	Cs-137	Normal thyroid cells	+	Nikiforova, Gandhi 2011
miR-193a	10Gy	4, 24h	Cs-137	Normal thyroid cells	+	Nikiforova, Gandhi 2011
miR-193b	10Gy	24h	Cs-137	B lymphoblasts IM9	+	Cha, Shin 2009
miR-194	2.5Gy	2, 24h	gamma	A549	+	Weidhaas, Babar 2007
miR-194	1Gy	6h	90 kV x-ray	Female mouse frontal lobe	-	Koturbash, Zemp
miR-194-5p	0.34, 1.3, 4.3, 13Gy	24h	^{177}Lu-octreotate	mouse renal cortical tissue	+	present study
miR-195	2Gy	1h	gamma	CD34+	-	Li, Ha 2012
miR-195	2.5Gy	2, 8h	gamma	A549	+	Weidhaas, Babar 2007
miR-195	1.25Gy	4h	gamma	human blood (TBI)	+	Templin, Paul 2011
miR-195-5p	6Gy	3h	Cs-137	differentiated kerotinocytes	+	Joly-Tonetti, Vinuelas
miR-195a-5p	13Gy	24h	^{177}Lu-octreotate	mouse renal cortical tissue	+	present study
miR-196a	6Gy	4h	6 MV photons	LNCaP prostate cancer cell	-	Josson, Sung 2008

miR-196a	6Gy	4h	6 MV photons	C4-2 prostate cancer cell	-	Josson, Sung 2008
miR-196a	8Gy	6h	gamma	HeLa	-	Hu, Tie 2013
miR-196a	2Gy	24h	Cs-137	human PBL	-	Girardi, De Pitta 2012
miR-196b	0.2Gy	4h	Cs-137	human PBL	+	Girardi, De Pitta 2012
miR-196b	2Gy	24h	Cs-137	human PBL	-	Girardi, De Pitta 2012
miR-196b-5p	6Gy	3h	Cs-137	differentiated kerotinocytes	+	Joly-Tonetti, Vinuelas
miR-197	2Gy	1h	gamma	CD34+	+	Li, Ha 2012
miR-197	8Gy	1h	gamma	hFOB	-	Li, Ha 2012
miR-197	2.5Gy	8, 24h	gamma	CRL2741	-	Weidhaas, Babar 2007
miR-197	2.5Gy	24h	gamma	A549	-	Weidhaas, Babar 2007
miR-197	0.05, 10Gy	8h	Cs-137	B lymphoblasts IM9	+	Cha, Seong 2009
miR-197	10Gy	24h	Cs-137	B lymphoblasts IM9	-	Cha, Shin 2009
miR-197-3p	6Gy	3h	Cs-137	differentiated kerotinocytes	+	Joly-Tonetti, Vinuelas
miR-197-3p	10 Gy	5, 15, 40h	gamma	PC3	-	Leung, Li 2014
miR-199a	6Gy	24h	15 MeV photons	prostate cancer cells LNCaP	-	Li, Shi 2011
miR-199a-2	0.05, 10Gy	8h	Cs-137	B lymphoblasts IM9	+	Cha, Seong 2009
miR-199a-3p	1.25Gy	4h	gamma	human blood (TBI)	+	Templin, Paul 2011
miR-199a-3p	0.34Gy	24h	^{177}Lu-octreotate	mouse renal cortical tissue	+	present study
miR-199a-5p	0.34, 4.3, 13Gy	24h	^{177}Lu-octreotate	mouse renal cortical tissue	+	present study
miR-199b-5p	0.2, 2Gy	4h	Cs-137	human PBL	+	Girardi, De Pitta 2012
miR-199b-5p	0.34, 1.3, 13Gy	24h	^{177}Lu-octreotate	mouse renal cortical tissue	+	present study
miR-200a	0.2Gy	0.5, 168h	gamma	human 3D tissue system	+-	Dickey, Zemp 2011
miR-200a	2Gy	0.5, 168h	gamma	human 3D tissue system	+-	Dickey, Zemp 2011
miR-200a	1.0 Gy	6,24h	p	mouse blood	-	Templin, Young 2012
miR-200a	2.5Gy	8, 24h	gamma	CRL2741	+	Weidhaas, Babar 2007
miR-200a	1Gy	6h	90 kV x-ray	Female mouse frontal lobe	+	Koturbash, Zemp
miR-200a-5p	2.5Gy	8, 24h	gamma	CRL2741	+	Weidhaas, Babar 2007
miR-200b	0.2Gy	0.5, 168h	gamma	human 3D tissue system	+-	Dickey, Zemp 2011
miR-200b	1.5Gy	6h	Cs-137	Mouse blood	-	Templin, Amundson 2011
miR-200b	2Gy	24h	Cs-137	human PBL	-	Girardi, De Pitta 2012
miR-200b	1Gy	6h	90 kV x-ray	Female mouse frontal lobe	+	Koturbash, Zemp
miR-200b-3p	6Gy	3h	Cs-137	proliferating kerotinocytes	-	Joly-Tonetti, Vinuelas
miR-200c	0.2Gy	168h	gamma	human 3D tissue system	-	Dickey, Zemp 2011

miR-200c	2Gy	168h	gamma	human 3D tissue system	+	Dickey, Zemp 2011
miR-200c	6Gy	24h	15 MeV photons	prostate cancer cells LNCaP	+	Li, Shi 2011
miR-200c	2.5Gy	2, 8, 24h	gamma	CRL2741	-	Weidhaas, Babar 2007
miR-200c	2.5Gy	6h	90 kV x-ray	Female mouse thymus	+	Ilnytskyy, Zemp 2008
miR-200c	1Gy	6h	90 kV x-ray	Female mouse frontal lobe	+	Koturbash, Zemp
miR-200c-3p	6Gy	3h	Cs-137	differentiated kerotinocytes	+	Joly-Tonetti, Vinuelas
miR-202	1, 10Gy	24h	Cs-137	B lymphoblasts IM9	-	Cha, Shin 2009
miR-202	0.2Gy	4h	Cs-137	human PBL	+	Girardi, De Pitta 2012
miR-202	0.2Gy	24h	Cs-137	human PBL	+	Girardi, De Pitta 2012
miR-203	0.2Gy	0.5, 168h	gamma	human 3D tissue system	-	Dickey, Zemp 2011
miR-203	2Gy	0.5h	gamma	human 3D tissue system	-	Dickey, Zemp 2011
miR-203	1.0 Gy	6 h	p	mouse blood	-	Templin, Young 2012
miR-203	1Gy	4, 24h	Cs-137	Normal thyroid cells	+	Nikiforova, Gandhi 2011
miR-203	10Gy	4, 24h	Cs-137	Normal thyroid cells	+	Nikiforova, Gandhi 2011
miR-204	1.0 Gy	6 h	proton	mouse blood	-	Templin, Young 2012
miR-204	8Gy	6h	gamma	HeLa	-	Hu, Tie 2013
miR-204	1Gy	6h	90 kV x-ray	Female mouse cerebellum	+	Koturbash, Zemp
miR-204-5p	4.3, 13Gy	24h	^{177}Lu-octreotate	mouse renal cortical tissue	+	present study
miR-205	0.2Gy	0.5, 48, 168h	gamma	human 3D tissue system	+-	Dickey, Zemp 2011
miR-205	2Gy	0.5, 48h	gamma	human 3D tissue system	+	Dickey, Zemp 2011
miR-205	2.5Gy	2, 24h	gamma	CRL2741	-	Weidhaas, Babar 2007
miR-205	2 Gy	4h	proton	Mouse brain	+	Khan, Tariq 2013
miR-205-3p	6Gy	3h	Cs-137	differentiated kerotinocytes	+	Joly-Tonetti, Vinuelas
miR-206	20, 40 Gy	24h	Cs-137	lung carcinoma cell A549	-	Shin, Cha 2009
miR-206	2.5Gy	24h	gamma	A549	+	Weidhaas, Babar 2007
miR-206	18.8Gy	4h	6 MV photon	U87MG glioblastoma	-	Chen, Zhu 2009
miR-206	2.5Gy	6h	90 kV x-ray	Female mouse spleen	+	Ilnytskyy, Zemp 2008
miR-206	1Gy	6h	90 kV x-ray	Male mouse hippocampus	+	Koturbash, Zemp
miR-207	0.05, 10Gy	8h	Cs-137	B lymphoblasts IM9	+	Cha, Seong 2009
miR-210	0.2, 2Gy	4h	Cs-137	human PBL	+	Girardi, De Pitta 2012
miR-210	2Gy	24h	Cs-137	human PBL	-	Girardi, De Pitta 2012
miR-211	5Gy	24h	Cs-137	Mouse blood	-	Templin, Amundson 2011
miR-211	0.5Gy	24h	Fe-56	Mouse blood	-	Templin, Amundson 2011

miR-211	1Gy	96h	90 kV x-ray	Female mouse hippocampus	+	Koturbash, Zemp
miR-214	2.5Gy	2, 8, 24h	gamma	A549	+-	Weidhaas, Babar 2007
miR-214	1Gy	6h	90 kV x-ray	Male mouse hippocampus	+	Koturbash, Zemp
miR-214-3p	0.34, 4.3Gy	24h	^{177}Lu-octreotate	mouse renal cortical tissue	+	present study
miR-215	40Gy	24h	Cs-137	lung carcinoma cell A549	+	Shin, Cha 2009
miR-216a	1Gy	6, 96h	90 kV x-ray	Male mouse cerebellum	+	Koturbash, Zemp
miR-216b	1Gy	6, 96h	90 kV x-ray	Female mouse cerebellum	+	Koturbash, Zemp
miR-216b	1Gy	6, 96h	90 kV x-ray	Male mouse cerebellum	+	Koturbash, Zemp
miR-217	1Gy	6h	90 kV x-ray	Female mouse cerebellum	+	Koturbash, Zemp
miR-217	1Gy	6h	90 kV x-ray	Male mouse cerebellum	+	Koturbash, Zemp
miR-218	1.0 Gy	6 h	p	mouse blood	-	Templin, Young 2012
miR-218	6Gy	4h	6 MV photons	C4-2 prostate cancer cell	-	Josson, Sung 2008
miR-219	1Gy	96h	90 kV x-ray	Female mouse hippocampus	+	Koturbash, Zemp
miR-220	0.05, 10Gy	8h	Cs-137	B lymphoblasts IM9	-	Cha, Seong 2009
miR-221	0.2, 2Gy	0.5, 168h	gamma	human 3D tissue system	+-	Dickey, Zemp 2011
miR-221	2.5Gy	2, 8, 24h	gamma	CRL2741	-	Weidhaas, Babar 2007
miR-221	2.5Gy	24h	gamma	A549	-	Weidhaas, Babar 2007
miR-221	1.25Gy	4h	gamma	human blood (TBI)	+	Templin, Paul 2011
miR-221	2.5Gy	6h	90 kV x-ray	Male mouse spleen	-	Ilnytskyy, Zemp 2008
miR-221	0.2Gy	4h	Cs-137	human PBL	+	Girardi, De Pitta 2012
miR-221-5p	2Gy	24h	Cs-137	human PBL	-	Girardi, De Pitta 2012
miR-221-5p	10 Gy	5, 15, 40h	gamma	PC3	-	Leung, Li 2014
miR-222	0.25, 0.5, 1, 3, 5, 10Gy	1h	Co-60	NorHuFib	-	Simone, Soule 2009
miR-222	0.2, 2Gy	0.5, 48, 168h	gamma	human 3D tissue system	+-	Dickey, Zemp 2011
miR-222	2.5Gy	24h	gamma	CRL2741	+	Weidhaas, Babar 2007
miR-222	2.5Gy	24h	gamma	A549	-	Weidhaas, Babar 2007
miR-222	1.25Gy	4h	gamma	human blood (TBI)	+	Templin, Paul 2011
miR-222	8Gy	6h	gamma	HeLa	+	Hu, Tie 2013
miR-222	1Gy	96h	90 kV x-ray	Female mouse cerebellum	-	Koturbash, Zemp
miR-222-3p	4.3Gy	24h	^{177}Lu-octreotate	mouse renal cortical tissue	+	present study
miR-223	2 Gy	0.5h	250 keV x-rays	foreskin fibroblasts	-	Maes, An 2008
miR-223	2Gy	1h	gamma	CD34+	+	Li, Ha 2012
miR-223	5Gy	6h	Cs-137	Mouse blood	+	Templin, Amundson 2011

miR-223	2.5Gy	6h	90 kV x-ray	Female mouse spleen	-	Ilnytskyy, Zemp 2008
miR-223	2Gy	4h	Cs-137	human PBL	+	Girardi, De Pitta 2012
miR-223	1Gy	96h	90 kV x-ray	Female mouse frontal lobe	-	Koturbash, Zemp
miR-224	0.2Gy	0.5, 168h	gamma	human 3D tissue system	-	Dickey, Zemp 2011
miR-224	2Gy	0.5, 168h	gamma	human 3D tissue system	-	Dickey, Zemp 2011
miR-224	1.0 Gy	6 h	p	mouse blood	-	Templin, Young 2012
miR-224	8Gy	1h	gamma	hFOB	+	Li, Ha 2012
miR-224	8Gy	6h	gamma	HeLa	-	Hu, Tie 2013
miR-224	2.5Gy	2, 8, 24h	gamma	A549	-	Weidhaas, Babar 2007
miR-290	2.5Gy	6h	90 kV x-ray	Female mouse spleen	+	Ilnytskyy, Zemp 2008
miR-292-39	0.5 Gy	6, 24h	p	mouse blood	-	Templin, Young 2012
miR-294	1.0 Gy	6 h	p	mouse blood	+	Templin, Young 2012
miR-294	2.5Gy	6h	90 kV x-ray	Male mouse spleen	+	Ilnytskyy, Zemp 2008
miR-298	1Gy	96h	90 kV x-ray	Female mouse frontal lobe	+	Koturbash, Zemp
miR-301	2.5Gy	8h	gamma	CRL2741	+	Weidhaas, Babar 2007
miR-301a	1.25Gy	4h	gamma	human blood (TBI)	+	Templin, Paul 2011
miR-301a	2Gy	4h	Cs-137	human PBL	+	Girardi, De Pitta 2012
miR-302b-5p	18.8Gy	4h	6 MV photon	U87MG glioblastoma	-	Chen, Zhu 2009
miR-320	0.2Gy	0.5, 168h	gamma	human 3D tissue system	-	Dickey, Zemp 2011
miR-320	2Gy	0.5, 168h	gamma	human 3D tissue system	+-	Dickey, Zemp 2011
miR-320	2.5Gy	8, 24h	gamma	CRL2741	-	Weidhaas, Babar 2007
miR-320	2.5Gy	2, 8, 24h	gamma	A549	-	Weidhaas, Babar 2007
miR-320a	8Gy	6h	gamma	HeLa	+	Hu, Tie 2013
miR-320a	8Gy	1h	gamma	hFOB	-	Li, Ha 2012
miR-320b	10 Gy	5, 15, 40h	gamma	PC3	-	Leung, Li 2014
miR-320c	8Gy	1h	gamma	hFOB	-	Li, Ha 2012
miR-320d	2Gy	1h	gamma	CD34+	-	Li, Ha 2012
miR-324-3p	2.5Gy	2h	gamma	A549	+	Weidhaas, Babar 2007
miR-324-3p	10Gy	8h	Cs-137	B lymphoblasts IM9	+	Cha, Seong 2009
miR-324-3p	2.5Gy	6h	90 kV x-ray	Female mouse spleen	+	Ilnytskyy, Zemp 2008
miR-324-3p	1Gy	96h	90 kV x-ray	Male mouse frontal lobe	-	Koturbash, Zemp
miR-324-5p	8Gy	1h	gamma	hFOB	-	Li, Ha 2012
miR-326	1Gy	4, 24h	Cs-137	Normal thyroid cells	-	Nikiforova, Gandhi 2011

miR-326	10Gy	4, 24h	Cs-137	Normal thyroid cells	-	Nikiforova, Gandhi 2011
miR-326	10Gy	8h	Cs-137	B lymphoblasts IM9	+	Cha, Seong 2009
miR-326	1Gy	96h	90 kV x-ray	Male mouse hippocampus	-	Koturbash, Zemp
miR-328	10Gy	8h	Cs-137	B lymphoblasts IM9	+	Cha, Seong 2009
miR-328	2.5Gy	6h	90 kV x-ray	Female mouse spleen	+	Ilnytskyy, Zemp 2008
miR-329	1Gy	6h	90 kV x-ray	Male mouse hippocampus	+	Koturbash, Zemp
miR-330-3p	2Gy	24h	Cs-137	human PBL	-	Girardi, De Pitta 2012
miR-331-3p	6Gy	3h	Cs-137	proliferating kerotinocytes	-	Joly-Tonetti, Vinuelas
miR-335	8Gy	1h	gamma	hFOB	+	Li, Ha 2012
miR-335	2.5Gy	2h	gamma	A549	+	Weidhaas, Babar 2007
miR-335	18.8Gy	4h	6 MV photon	U87MG glioblastoma	+	Chen, Zhu 2009
miR-335	2Gy	24h	Cs-137	human PBL	-	Girardi, De Pitta 2012
miR-335-3p	1.5, 5Gy	24h	Cs-137	Mouse blood	+	Templin, Amundson 2011
miR-337-3p	5Gy	24h	Cs-137	Mouse blood	-	Templin, Amundson 2011
miR-338	1Gy	4, 24h	Cs-137	Normal thyroid cells	-	Nikiforova, Gandhi 2011
miR-338	10Gy	4, 24h	Cs-137	Normal thyroid cells	-	Nikiforova, Gandhi 2011
miR-339	10Gy	24h	Cs-137	B lymphoblasts IM9	+	Cha, Shin 2009
miR-339-3p	5Gy	24h	Cs-137	Mouse blood	+	Templin, Amundson 2011
miR-339-3p	0.2Gy	4h	Cs-137	human PBL	-	Girardi, De Pitta 2012
miR-340	1.25Gy	4h	gamma	human blood (TBI)	+	Templin, Paul 2011
miR-341	1Gy	96h	90 kV x-ray	Female mouse frontal lobe	+	Koturbash, Zemp
miR-342	10Gy	24h	Cs-137	B lymphoblasts IM9	+	Cha, Shin 2009
mir-342-3p	6Gy	3h	Cs-137	differentiated kerotinocytes	+	Joly-Tonetti, Vinuelas
mir-342-3p	10mGy	3h	Cs-137	proliferating kerotinocytes	+	Joly-Tonetti, Vinuelas
mir-342-3p	5Gy	24h	Cs-137	Mouse blood	-	Templin, Amundson 2011
mir-342-3p	0.5Gy	24h	Fe-56	Mouse blood	-	Templin, Amundson 2011
mir-342-3p	0.34Gy	24h	^{177}Lu-octreotate	mouse renal cortical tissue	+	present study
mir-342-5p	10 Gy	5, 15, 40h	gamma	PC3	-	Leung, Li 2014
miR-345	20, 40Gy	24h	Cs-137	lung carcinoma cell A549	-	Shin, Cha 2009
miR-345	0.2Gy	4h	Cs-137	human PBL	-	Girardi, De Pitta 2012
miR-345	2Gy	24h	Cs-137	human PBL	+	Girardi, De Pitta 2012
miR-346	2.5Gy	6h	90 kV x-ray	Male mouse spleen	+	Ilnytskyy, Zemp 2008
miR-346	2.5Gy	6h	90 kV x-ray	Female mouse spleen	+	Ilnytskyy, Zemp 2008

miR-346	2.5Gy	6h	90 kV x-ray	Male mouse thymus	-	Ilnytskyy, Zemp 2008
miR-346	1Gy	96h	90 kV x-ray	Male mouse frontal lobe	-	Koturbash, Zemp
miR-350	0.1Gy	6h	Fe-56	Mouse blood	-	Templin, Amundson 2011
miR-361	2.5Gy	2, 8, 24h	gamma	CRL2741	-	Weidhaas, Babar 2007
miR-361	8Gy	6h	gamma	HeLa	+	Hu, Tie 2013
miR-361	2.5Gy	2, 8, 24h	gamma	A549	-	Weidhaas, Babar 2007
miR-361-3p	10 Gy	5, 15, 40h	gamma	PC3	-	Leung, Li 2014
miR-361-5p	0.34, 1.3, 4.3Gy	24h	^{177}Lu-octreotate	mouse renal cortical tissue	+	present study
miR-362-5p	1.0 Gy	24 h	p	mouse blood	-	Templin, Young 2012
miR-362-5p	1.25Gy	4h	gamma	human blood (TBI)	+	Templin, Paul 2011
miR-363	2Gy	24h	Cs-137	human PBL	+	Girardi, De Pitta 2012
miR-363-3p	2.5Gy	6h	90 kV x-ray	Male mouse spleen	-	Ilnytskyy, Zemp 2008
miR-365	1Gy	24h	Cs-137	Normal thyroid cells	-	Nikiforova, Gandhi 2011
miR-365	2.5Gy	2, 8, 24h	gamma	A549	+	Weidhaas, Babar 2007
miR-365	8Gy	6h	gamma	HeLa	-	Hu, Tie 2013
miR-365	10Gy	24h	Cs-137	B lymphoblasts IM9	+	Cha, Shin 2009
miR-365-3p	0.13Gy	24h	^{177}Lu-octreotate	mouse renal cortical tissue	-	present study
miR-368	2.5Gy	2h	gamma	A549	+	Weidhaas, Babar 2007
miR-371-5p	2Gy	24h	Cs-137	human PBL	+	Girardi, De Pitta 2012
miR-372	6Gy	4h	6 MV photons	LNCaP prostate cancer cell	+	Josson, Sung 2008
miR-373	8Gy	1h	gamma	hFOB	+	Li, Ha 2012
miR-374	2 Gy	4h	proton	Mouse liver	+	Khan, Tariq 2013
miR-374a	2.5Gy	2, 8h	gamma	A549	+	Weidhaas, Babar 2007
miR-374a	1.25Gy	4h	gamma	human blood (TBI)	+	Templin, Paul 2011
miR-374a-5p	10 Gy	5, 15, 40h	gamma	PC3	-	Leung, Li 2014
miR-375	1Gy	96h	90 kV x-ray	Female mouse cerebellum	-	Koturbash, Zemp
miR-376a	40Gy	24h	Cs-137	lung carcinoma cell A549	+	Shin, Cha 2009
miR-376a	0.1 Gy	24h	250 keV x-rays	foreskin fibroblasts	-	Maes, An 2008
miR-376a	1.25Gy	4h	gamma	human blood (TBI)	+	Templin, Paul 2011
miR-376a	0.05Gy	8h	Cs-137	B lymphoblasts IM9	-	Cha, Seong 2009
miR-377	1Gy	4h	Cs-137	Normal thyroid cells	-	Nikiforova, Gandhi 2011
miR-377	10Gy	4h	Cs-137	Normal thyroid cells	-	Nikiforova, Gandhi 2011
miR-378	0.2, 2Gy	4h	Cs-137	human PBL	-	Girardi, De Pitta 2012

miR-378a-3p	0.34	24h	^{177}Lu-octreotate	mouse renal cortical tissue	+	present study
miR-378c	0.34	24h	^{177}Lu-octreotate	mouse renal cortical tissue	+	present study
miR-379	1.0 Gy	6 h	p	mouse blood	-	Templin, Young 2012
miR-379	0.5, 1Gy	24 h	p	mouse blood	-	Templin, Young 2012
miR-379	6Gy	4h	6 MV photons	C4-2 prostate cancer cell	+	Josson, Sung 2008
miR-379	0.1Gy	6h	Fe-56	Mouse blood	+	Templin, Amundson 2011
miR-379	18.8Gy	4h	6 MV photon	U87MG glioblastoma	+	Chen, Zhu 2009
miR-380-5p	1.0 Gy	6 h	p	mouse blood	-	Templin, Young 2012
miR-382	8Gy	1h	gamma	hFOB	+	Li, Ha 2012
miR-382	1Gy	6, 96h	90 kV x-ray	Female mouse frontal lobe	+	Koturbash, Zemp
miR-383	6Gy	4h	6 MV photons	C4-2 prostate cancer cell	+	Josson, Sung 2008
miR-383	0.1, 0.5Gy	6h	Fe-56	Mouse blood	-	Templin, Amundson 2011
miR-384	1Gy	4, 24h	Cs-137	Normal thyroid cells	-	Nikiforova, Gandhi 2011
miR-384	10Gy	4, 24h	Cs-137	Normal thyroid cells	-	Nikiforova, Gandhi 2011
miR-384	1Gy	96h	90 kV x-ray	Female mouse hippocampus	+	Koturbash, Zemp
miR-409-3p	0.1Gy	24h	Fe-56	Mouse blood	+	Templin, Amundson 2011
miR-409-5p	1Gy	4, 24h	Cs-137	Normal thyroid cells	+-	Nikiforova, Gandhi 2011
miR-409-5p	10Gy	4, 24h	Cs-137	Normal thyroid cells	+-	Nikiforova, Gandhi 2011
miR-409-5p	2 Gy	4h	proton	Mouse brain	+	Khan, Tariq 2013
miR-409-5p	2 Gy	4h	proton	Mouse testis	+	Khan, Tariq 2013
miR-411	1.0 Gy	6 h	p	mouse blood	-	Templin, Young 2012
miR-411	1Gy	96h	90 kV x-ray	Female mouse hippocampus	+	Koturbash, Zemp
miR-412-5p	2 Gy	4h	proton	Mouse brain	+	Khan, Tariq 2013
miR-421	0.1 Gy	0.5h	250 keV x-rays	foreskin fibroblasts	-	Maes, An 2008
miR-421	2Gy	24h	Cs-137	human PBL	+	Girardi, De Pitta 2012
miR-423-3p	2 Gy	4h	proton	Mouse testis	+	Khan, Tariq 2013
miR-423-5p	2Gy	168h	gamma	human 3D tissue system	+	Dickey, Zemp 2011
miR-424	2.5Gy	2, 8, 24h	gamma	CRL2741	-	Weidhaas, Babar 2007
miR-424	2.5Gy	2, 8, 24h	gamma	A549	-	Weidhaas, Babar 2007
miR-424-3p	0.2, 2Gy	24h	Cs-137	human PBL	+	Girardi, De Pitta 2012
miR-425	0.2, 2Gy	0.5, 48, 168h	gamma	human 3D tissue system	+	Dickey, Zemp 2011
miR-425-3p	0.2Gy	4h	Cs-137	human PBL	+	Girardi, De Pitta 2012
miR-425-5p	1.3, 4.3Gy	24h	^{177}Lu-octreotate	mouse renal cortical tissue	+	present study

miR-429	2.5Gy	8, 24h	gamma	CRL2741	+	Weidhaas, Babar 2007
miR-429	1Gy	6h	90 kV x-ray	Female mouse frontal lobe	+	Koturbash, Zemp
miR-432	10Gy	24h	Cs-137	B lymphoblasts IM9	-	Cha, Shin 2009
miR-434-3p	1Gy	6h	90 kV x-ray	Male mouse frontal lobe	+	Koturbash, Zemp
miR-449	6Gy	4h	6 MV photons	LNCaP prostate cancer cell	+	Josson, Sung 2008
miR-449a	0.2Gy	0.5, 48h	gamma	human 3D tissue system	+	Dickey, Zemp 2011
miR-449a	2Gy	0.5, 48, 168h	gamma	human 3D tissue system	+-	Dickey, Zemp 2011
miR-449b	0.2Gy	0.5, 48, 168h	gamma	human 3D tissue system	+	Dickey, Zemp 2011
miR-449b	2Gy	0.5, 48, 168h	gamma	human 3D tissue system	+-	Dickey, Zemp 2011
miR-450	2.5Gy	2, 24h	gamma	A549	+	Weidhaas, Babar 2007
miR-450a	0.2Gy	4h	Cs-137	human PBL	+	Girardi, De Pitta 2012
miR-450a-5p	5Gy	6h	Cs-137	Mouse blood	+	Templin, Amundson 2011
miR-451	18.8Gy	4h	6 MV photon	U87MG glioblastoma	+	Chen, Zhu 2009
miR-451	1Gy	6h	90 kV x-ray	Female mouse frontal lobe	-	Koturbash, Zemp
miR-451	2 Gy	4h	proton	Mouse testis	-	Khan, Tariq 2013
miR-451a	4.3Gy	24h	^{177}Lu-octreotate	mouse renal cortical tissue	+	present study
miR-452	1Gy	24h	Cs-137	Normal thyroid cells	+	Nikiforova, Gandhi 2011
miR-452	10Gy	4, 24h	Cs-137	Normal thyroid cells	+-	Nikiforova, Gandhi 2011
miR-452-3p	2.5Gy	2h	gamma	A549	+	Weidhaas, Babar 2007
miR-453	1Gy	4, 24h	Cs-137	Normal thyroid cells	+-	Nikiforova, Gandhi 2011
miR-453	10Gy	4, 24h	Cs-137	Normal thyroid cells	+-	Nikiforova, Gandhi 2011
miR-454	2Gy	1h	gamma	CD34+	-	Li, Ha 2012
miR-454	1.25Gy	4h	gamma	human blood (TBI)	+	Templin, Paul 2011
miR-455-3p	1Gy	6, 96h	90 kV x-ray	Male mouse frontal lobe	-	Koturbash, Zemp
mir-466	8Gy	1h	gamma	hFOB	+	Li, Ha 2012
miR-467a-5p	1Gy	96h	90 kV x-ray	Female mouse hippocampus	+	Koturbash, Zemp
miR-467b	2.5Gy	6h	90 kV x-ray	Male mouse thymus	-	Ilnytskyy, Zemp 2008
miR-467d	2 Gy	4h	proton	Mouse testis	-	Khan, Tariq 2013
miR-483	1Gy	96h	90 kV x-ray	Female mouse cerebellum	+	Koturbash, Zemp
miR-483-5p	2Gy	24h	Cs-137	human PBL	+	Girardi, De Pitta 2012
miR-484	0.34, 1.3Gy	24h	^{177}Lu-octreotate	mouse renal cortical tissue	+	present study
miR-485-3p	8Gy	1h	gamma	hFOB	+	Li, Ha 2012
miR-486-5p	2Gy	1h	gamma	CD34+	+	Li, Ha 2012

miR-487	6Gy	4h	6 MV photons	LNCaP prostate cancer cell	-	Josson, Sung 2008
miR-488	6Gy	4h	6 MV photons	C4-2 prostate cancer cell	+	Josson, Sung 2008
miR-488	1Gy	96h	90 kV x-ray	Female mouse hippocampus	+	Koturbash, Zemp
miR-488-5p	1Gy	6h	90 kV x-ray	Male mouse hippocampus	-	Koturbash, Zemp
miR-488-5p	1Gy	6, 96h	90 kV x-ray	Male mouse cerebellum	+	Koturbash, Zemp
miR-489	1Gy	4, 24	Cs-137	Normal thyroid cells	+	Nikiforova, Gandhi 2011
miR-489	10Gy	4, 24	Cs-137	Normal thyroid cells	+	Nikiforova, Gandhi 2011
miR-489	8Gy	1h	gamma	hFOB	+	Li, Ha 2012
miR-493	8Gy	1h	gamma	hFOB	+	Li, Ha 2012
miR-494	0.5Gy	24h	Fe-56	Mouse blood	-	Templin, Amundson 2011
miR-494	0.2Gy	4h	Cs-137	human PBL	-	Girardi, De Pitta 2012
miR-494	2Gy	24h	Cs-137	human PBL	+	Girardi, De Pitta 2012
miR-495	0.5 Gy	6 h	p	mouse blood	-	Templin, Young 2012
miR-497	2 Gy	4h	proton	Mouse testis	-	Khan, Tariq 2013
miR-500	18.8Gy	4h	6 MV photon	U87MG glioblastoma	+	Chen, Zhu 2009
miR-501-3p	0.5 Gy	6 h	p	mouse blood	+	Templin, Young 2012
miR-501-3p	5Gy	24h	Cs-137	Mouse blood	+	Templin, Amundson 2011
miR-501-3p	0.5Gy	6h	Fe-56	Mouse blood	+	Templin, Amundson 2011
miR-501-3p	2 Gy	4h	proton	Mouse brain	+	Khan, Tariq 2013
miR-501-3p	2 Gy	4h	proton	Mouse testis	+	Khan, Tariq 2013
miR-501-3p	10 Gy	5, 15, 40h	gamma	PC3	-	Leung, Li 2014
miR-502-3p	2Gy	1h	gamma	CD34+	-	Li, Ha 2012
miR-502-5p	1.25Gy	4h	gamma	human blood (TBI)	+	Templin, Paul 2011
miR-503	2 Gy	6h	6MV gamma	endoth cells	-	Wagner-Ecker, Schwager 2010
miR-503	2.5Gy	8, 24h	gamma	CRL2741	-	Weidhaas, Babar 2007
miR-503	2.5Gy	2h	gamma	A549	-	Weidhaas, Babar 2007
miR-505	8Gy	1h	gamma	hFOB	-	Li, Ha 2012
miR-505-5p	2Gy	24h	Cs-137	human PBL	-	Girardi, De Pitta 2012
miR-511	1.0 Gy	6 h	p	mouse blood	+	Templin, Young 2012
miR-511	0.5Gy	6h	Cs-137	Mouse blood	+	Templin, Amundson 2011
miR-511	5Gy	24h	Cs-137	Mouse blood	+	Templin, Amundson 2011
miR-513a-5p	2Gy	4h	Cs-137	human PBL	-	Girardi, De Pitta 2012
miR-513a-5p	2Gy	24h	Cs-137	human PBL	+	Girardi, De Pitta 2012

miR-513b	0.2, 2Gy	4h	Cs-137	human PBL	-	Girardi, De Pitta 2012
miR-513b	2Gy	24h	Cs-137	human PBL	+	Girardi, De Pitta 2012
miR-513c	2Gy	24h	Cs-137	human PBL	+	Girardi, De Pitta 2012
miR-516a-5p	20, 40Gy	24h	Cs-137	lung carcinoma cell A549	-	Shin, Cha 2009
miR-517c	2 Gy	0.5h	250 keV x-rays	foreskin fibroblasts	-	Maes, An 2008
miR-518b	18.8Gy	4h	6 MV photon	U87MG glioblastoma	-	Chen, Zhu 2009
miR-520a	1Gy	4, 24h	Cs-137	Normal thyroid cells	+	Nikiforova, Gandhi 2011
miR-520a	10Gy	4, 24h	Cs-137	Normal thyroid cells	+	Nikiforova, Gandhi 2011
miR-520b	1, 10Gy	24h	Cs-137	B lymphoblasts IM9	+	Cha, Shin 2009
miR-520c	6Gy	4h	6 MV photons	LNCaP prostate cancer cell	+	Josson, Sung 2008
miR-520d	2 Gy	0.5h	250 keV x-rays	foreskin fibroblasts	-	Maes, An 2008
miR-520d	1, 10Gy	24h	Cs-137	B lymphoblasts IM9	-	Cha, Shin 2009
miR-520e	1Gy	4, 24h	Cs-137	Normal thyroid cells	+-	Nikiforova, Gandhi 2011
miR-520e	10Gy	4h	Cs-137	Normal thyroid cells	-	Nikiforova, Gandhi 2011
miR-520f	6Gy	4h	6 MV photons	LNCaP prostate cancer cell	+	Josson, Sung 2008
miR-521	6Gy	4h	6 MV photons	LNCaP prostate cancer cell	-	Josson, Sung 2008
miR-521	6Gy	4h	6 MV photons	C4-2 prostate cancer cell	-	Josson, Sung 2008
miR-521	18.8Gy	4h	6 MV photon	U87MG glioblastoma	-	Chen, Zhu 2009
mir-522	1Gy	4, 24h	Cs-137	Normal thyroid cells	-	Nikiforova, Gandhi 2011
mir-522	10Gy	4, 24h	Cs-137	Normal thyroid cells	-	Nikiforova, Gandhi 2011
miR-526b	1Gy	4, 24h	Cs-137	Normal thyroid cells	+-	Nikiforova, Gandhi 2011
miR-526b	10Gy	4, 24h	Cs-137	Normal thyroid cells	+-	Nikiforova, Gandhi 2011
miR-532-5p	2Gy	1h	gamma	CD34+	-	Li, Ha 2012
miR-539	10Gy	24h	Cs-137	B lymphoblasts IM9	+	Cha, Shin 2009
miR-542-5p	2.5Gy	8, 24h	gamma	CRL2741	-	Weidhaas, Babar 2007
miR-543	1Gy	6h	90 kV x-ray	Female mouse frontal lobe	-	Koturbash, Zemp
miR-543	1Gy	96h	90 kV x-ray	Female mouse frontal lobe	-	Koturbash, Zemp
miR-547	1.5Gy	6h	Cs-137	Mouse blood	-	Templin, Amundson 2011
miR-548b-3p	2 Gy	2h	250 keV x-rays	foreskin fibroblasts	+	Maes, An 2008
miR-548c-3p	20, 40Gy	24h	Cs-137	lung carcinoma cell A549	-	Shin, Cha 2009
miR-548d	18.8Gy	4h	6 MV photon	U87MG glioblastoma	+	Chen, Zhu 2009
miR-548d-3p	1.25Gy	4h	gamma	human blood (TBI)	+	Templin, Paul 2011
miR-548h-5p	10 Gy	5, 15, 40h	gamma	PC3	+	Leung, Li 2014

miR-550a-3p	10 Gy	5, 15, 40h	gamma	PC3	+	Leung, Li 2014
miR-551b	2Gy	24h	Cs-137	human PBL	-	Girardi, De Pitta 2012
miR-557	1, 10Gy	24h	Cs-137	B lymphoblasts IM9	+	Cha, Shin 2009
miR-558	2 Gy	2h	250 keV x-rays	foreskin fibroblasts	+	Maes, An 2008
miR-560	1, 10Gy	24h	Cs-137	B lymphoblasts IM9	+	Cha, Shin 2009
miR-564	2 Gy	0.5h	250 keV x-rays	foreskin fibroblasts	-	Maes, An 2008
miR-564	1Gy	24h	Cs-137	B lymphoblasts IM9	+	Cha, Shin 2009
miR-565	2.5Gy	2, 8h	gamma	A549	+	Weidhaas, Babar 2007
miR-570	1.25Gy	4h	gamma	human blood (TBI)	+	Templin, Paul 2011
miR-572	1, 10Gy	24h	Cs-137	B lymphoblasts IM9	+	Cha, Shin 2009
miR-574-3p	0.2Gy	0.5h	gamma	human 3D tissue system	-	Dickey, Zemp 2011
miR-574-3p	2Gy	0.5, 48h	gamma	human 3D tissue system	-	Dickey, Zemp 2011
miR-574-5p	0.2Gy	0.5, 48h	gamma	human 3D tissue system	-	Dickey, Zemp 2011
miR-574-5p	2Gy	0.5, 48h	gamma	human 3D tissue system	-	Dickey, Zemp 2011
miR-574-5p	2Gy	24h	Cs-137	human PBL	+	Girardi, De Pitta 2012
miR-575	2Gy	1h	gamma	CD34+	-	Li, Ha 2012
miR-575	1, 10Gy	24h	Cs-137	B lymphoblasts IM9	+	Cha, Shin 2009
miR-575	2Gy	4h	Cs-137	human PBL	-	Girardi, De Pitta 2012
miR-579	2 Gy	0.5h	250 keV x-rays	foreskin fibroblasts	-	Maes, An 2008
miR-582	2.5Gy	2, 8, 24h	gamma	A549	+	Weidhaas, Babar 2007
miR-582	18.8Gy	4h	6 MV photon	U87MG glioblastoma	-	Chen, Zhu 2009
miR-582-5p	2 Gy	2h	250 keV x-rays	foreskin fibroblasts	+	Maes, An 2008
miR-582-5p	0.2, 2Gy	4h	Cs-137	human PBL	+	Girardi, De Pitta 2012
miR-583	1, 10Gy	24h	Cs-137	B lymphoblasts IM9	+	Cha, Shin 2009
miR-584	2.5Gy	24h	gamma	CRL2741	-	Weidhaas, Babar 2007
miR-585	2 Gy	2h	250 keV x-rays	foreskin fibroblasts	+	Maes, An 2008
miR-592	1Gy	96h	90 kV x-ray	Female mouse hippocampus	+	Koturbash, Zemp
miR-593	20 Gy	24h	Cs-137	lung carcinoma cell A549	-	Shin, Cha 2009
miR-594	2.5Gy	2, 8, 24h	gamma	CRL2741	-	Weidhaas, Babar 2007
miR-594	2.5Gy	2, 24h	gamma	A549	-	Weidhaas, Babar 2007
miR-595	10Gy	24h	Cs-137	B lymphoblasts IM9	+	Cha, Shin 2009
miR-598	1.0 Gy	6, 24h	p	mouse blood	-	Templin, Young 2012
miR-598	0.1 Gy	24h	250 keV x-rays	foreskin fibroblasts	-	Maes, An 2008

miR-598	1.25Gy	4h	gamma	human blood (TBI)	+	Templin, Paul 2011
miR-598	0.5Gy	6h	Cs-137	Mouse blood	+	Templin, Amundson 2011
miR-601	18.8Gy	4h	6 MV photon	U87MG glioblastoma	+	Chen, Zhu 2009
miR-601	0.2Gy	24h	Cs-137	human PBL	+	Girardi, De Pitta 2012
miR-605	2Gy	1h	gamma	CD34+	-	Li, Ha 2012
miR-605	2.5Gy	8h	gamma	CRL2741	-	Weidhaas, Babar 2007
miR-608	2 Gy	0.5h	250 keV x-rays	foreskin fibroblasts	-	Maes, An 2008
miR-609	2 Gy	0.5h	250 keV x-rays	foreskin fibroblasts	-	Maes, An 2008
miR-609	2 Gy	6h	250 keV x-rays	foreskin fibroblasts	-	Maes, An 2008
miR-612	2.5Gy	8h	gamma	A549	+	Weidhaas, Babar 2007
miR-617	2 Gy	0.5h	250 keV x-rays	foreskin fibroblasts	-	Maes, An 2008
miR-617	1Gy	24h	Cs-137	B lymphoblasts IM9	+	Cha, Shin 2009
miR-620	18.8Gy	4h	6 MV photon	U87MG glioblastoma	-	Chen, Zhu 2009
miR-623	2 Gy	0.5h	250 keV x-rays	foreskin fibroblasts	-	Maes, An 2008
miR-625	8Gy	1h	gamma	hFOB	+	Li, Ha 2012
miR-628-3p	8Gy	1h	gamma	hFOB	+	Li, Ha 2012
miR-629-3p	0.2Gy	4h	Cs-137	human PBL	+	Girardi, De Pitta 2012
miR-630	1, 10Gy	24h	Cs-137	B lymphoblasts IM9	+	Cha, Shin 2009
miR-630	0.2, 2Gy	4h	Cs-137	human PBL	-	Girardi, De Pitta 2012
miR-630	2Gy	24h	Cs-137	human PBL	+	Girardi, De Pitta 2012
miR-636	20 Gy	24h	Cs-137	lung carcinoma cell A549	-	Shin, Cha 2009
miR-638	0.25, 0.5, 1, 3, 5, 10Gy	1h	Co-60	NorHuFib	-	Simone, Soule 2009
miR-638	2Gy	1h	gamma	CD34+	-	Li, Ha 2012
miR-638	2.5Gy	2, 8, 24h	gamma	CRL2741	+-	Weidhaas, Babar 2007
miR-638	2.5Gy	2, 8h	gamma	A549	+-	Weidhaas, Babar 2007
miR-638	1, 10Gy	24h	Cs-137	B lymphoblasts IM9	+	Cha, Shin 2009
miR-638	0.2, 2Gy	24h	Cs-137	human PBL	+	Girardi, De Pitta 2012
miR-641	18.8Gy	4h	6 MV photon	U87MG glioblastoma	+	Chen, Zhu 2009
miR-646	18.8Gy	4h	6 MV photon	U87MG glioblastoma	+	Chen, Zhu 2009
miR-648	0.1 Gy	0.5h	250 keV x-rays	foreskin fibroblasts	-	Maes, An 2008
miR-654	10Gy	24h	Cs-137	B lymphoblasts IM9	+	Cha, Shin 2009
miR-656	0.1 Gy	0.5h	250 keV x-rays	foreskin fibroblasts	-	Maes, An 2008
miR-659	0.1 Gy	24h	250 keV x-rays	foreskin fibroblasts	-	Maes, An 2008

miR-660	2 Gy	24h	250 keV x-rays	foreskin fibroblasts	-	Maes, An 2008
miR-660	1.25Gy	4h	gamma	human blood (TBI)	+	Templin, Paul 2011
miR-662	2 Gy	2h	250 keV x-rays	foreskin fibroblasts	+	Maes, An 2008
miR-663	0.25, 0.5, 1, 3, 5, 10Gy	1h	Co-60	NorHuFib	+	Simone, Soule 2009
miR-663	2 Gy	0.5h	250 keV x-rays	foreskin fibroblasts	-	Maes, An 2008
miR-663	2.5Gy	24h	gamma	CRL2741	+	Weidhaas, Babar 2007
miR-663	2.5Gy	8, 24h	gamma	A549	+	Weidhaas, Babar 2007
miR-663	1, 10Gy	24h	Cs-137	B lymphoblasts IM9	+	Cha, Shin 2009
miR-663	0.2, 2Gy	24h	Cs-137	human PBL	+	Girardi, De Pitta 2012
miR-667	0.5 Gy	6 h	p	mouse blood	-	Templin, Young 2012
miR-667	1.0 Gy	24 h	p	mouse blood	+	Templin, Young 2012
miR-667	1.5, 5Gy	24h	Cs-137	Mouse blood	-	Templin, Amundson 2011
miR-667	1Gy	6h	90 kV x-ray	Female mouse cerebellum	-	Koturbash, Zemp
miR-671	1Gy	24h	Cs-137	B lymphoblasts IM9	+	Cha, Shin 2009
miR-671-3p	10 Gy	5, 15, 40h	gamma	PC3	-	Leung, Li 2014
miR-674	2 Gy	4h	proton	Mouse brain	+	Khan, Tariq 2013
miR-676-5p	1Gy	6h	90 kV x-ray	Female mouse frontal lobe	-	Koturbash, Zemp
miR-677-3p	1.3Gy	24h	^{177}Lu-octreotate	mouse renal cortical tissue	+	present study
miR-678	2.5Gy	6h	90 kV x-ray	Male mouse thymus	-	Ilnytskyy, Zemp 2008
miR-680	1.5, 5Gy	6h	Cs-137	Mouse blood	+	Templin, Amundson 2011
miR-680	0.5Gy	6h	Fe-56	Mouse blood	+	Templin, Amundson 2011
miR-685	1.0 Gy	24 h	p	mouse blood	+	Templin, Young 2012
miR-685	1.5, 5Gy	6h	Cs-137	Mouse blood	+	Templin, Amundson 2011
miR-685	0.1Gy	24h	Fe-56	Mouse blood	+	Templin, Amundson 2011
miR-685	1Gy	96h	90 kV x-ray	Female mouse frontal lobe	+	Koturbash, Zemp
miR-689	2.5Gy	6h	90 kV x-ray	Male mouse spleen	+	Ilnytskyy, Zemp 2008
miR-690	13Gy	24h	^{177}Lu-octreotate	mouse renal cortical tissue	-	present study
miR-690	2 Gy	4h	proton	Mouse liver	+	Khan, Tariq 2013
miR-702	1Gy	96h	90 kV x-ray	Female mouse hippocampus	-	Koturbash, Zemp
miR-704	1Gy	96h	90 kV x-ray	Female mouse cerebellum	-	Koturbash, Zemp
miR-705	2.5Gy	6h	90 kV x-ray	Male mouse thymus	-	Ilnytskyy, Zemp 2008
miR-706	2.5Gy	6h	90 kV x-ray	Male mouse thymus	-	Ilnytskyy, Zemp 2008
miR-708	1.5Gy	6h	Cs-137	Mouse blood	-	Templin, Amundson 2011

miR-708	1Gy	96h	90 kV x-ray	Male mouse frontal lobe	-	Koturbash, Zemp
miR-708-5p	6Gy	3h	Cs-137	proliferating kerotinocytes	-	Joly-Tonetti, Vinuelas
miR-708-5p	10mGy	3h	Cs-137	proliferating kerotinocytes	+	Joly-Tonetti, Vinuelas
miR-709	13Gy	24h	^{177}Lu-octreotate	mouse renal cortical tissue	-	present study
miR-711	2.5Gy	6h	90 kV x-ray	Male mouse thymus	-	Ilnytskyy, Zemp 2008
miR-712	2 Gy	4h	proton	Mouse testis	-	Khan, Tariq 2013
miR-715	1Gy	96h	90 kV x-ray	Female mouse cerebellum	+	Koturbash, Zemp
miR-718	8Gy	1h	gamma	hFOB	-	Li, Ha 2012
miR-720	2Gy	1h	gamma	CD34+	+	Li, Ha 2012
miR-720	1Gy	96h	90 kV x-ray	Male mouse cerebellum	+	Koturbash, Zemp
miR-720	1Gy	6, 96h	90 kV x-ray	Female mouse frontal lobe	+	Koturbash, Zemp
miR-720	2 Gy	4h	proton	Mouse testis	+	Khan, Tariq 2013
miR-741	1.0 Gy	24 h	p	mouse blood	+	Templin, Young 2012
miR-760	20 Gy	24h	Cs-137	lung carcinoma cell A549	-	Shin, Cha 2009
miR-760	0.2Gy	24h	Cs-137	human PBL	+	Girardi, De Pitta 2012
miR-765	8Gy	1h	gamma	hFOB	+	Li, Ha 2012
miR-765	0.2, 2Gy	24h	Cs-137	human PBL	+	Girardi, De Pitta 2012
mir-766	2Gy	1h	gamma	CD34+	+	Li, Ha 2012
miR-768-3p	0.25, 0.5, 1, 3, 5, 10Gy	1h	Co-60	NorHuFib	+	Simone, Soule 2009
miR-768-5p	0.25, 0.5, 1, 3, 5, 10Gy	1h	Co-60	NorHuFib	+	Simone, Soule 2009
miR-768-5p	2Gy	4h	Cs-137	human PBL	-	Girardi, De Pitta 2012
miR-769-5p	2Gy	24h	Cs-137	human PBL	-	Girardi, De Pitta 2012
miR-801	0.2Gy	4h	Cs-137	human PBL	-	Girardi, De Pitta 2012
miR-801	2Gy	24h	Cs-137	human PBL	+	Girardi, De Pitta 2012
miR-872	2 Gy	4h	proton	Mouse testis	-	Khan, Tariq 2013
miR-873	2Gy	24h	Cs-137	human PBL	-	Girardi, De Pitta 2012
miR-877-3p	2Gy	24h	Cs-137	human PBL	+	Girardi, De Pitta 2012
miR-879	0.5Gy	24h	Fe-56	Mouse blood	+	Templin, Amundson 2011
miR-885-3p	20, 40Gy	24h	Cs-137	lung carcinoma cell A549	-	Shin, Cha 2009
miR-886-3p	0.2, 2Gy	4h	Cs-137	human PBL	-	Girardi, De Pitta 2012
miR-886-3p	0.2Gy	24h	Cs-137	human PBL	-	Girardi, De Pitta 2012
miR-923	0.2, 2Gy	4h	Cs-137	human PBL	-	Girardi, De Pitta 2012
miR-923	2Gy	24h	Cs-137	human PBL	+	Girardi, De Pitta 2012

miR-940	2Gy	4h	Cs-137	human PBL	+	Girardi, De Pitta 2012
miR-940	2Gy	24h	Cs-137	human PBL	+	Girardi, De Pitta 2012
miR-1225-5p	0.2, 2Gy	24h	Cs-137	human PBL	+	Girardi, De Pitta 2012
miR-1226-5p	0.2Gy	24h	Cs-137	human PBL	+	Girardi, De Pitta 2012
miR-1228	40Gy	24h	Cs-137	lung carcinoma cell A549	-	Shin, Cha 2009
miR-1247	2 Gy	4h	proton	Mouse testis	-	Khan, Tariq 2013
miR-1260b	2Gy	1h	gamma	CD34+	+	Li, Ha 2012
mir-1275	2Gy	1h	gamma	CD34+	-	Li, Ha 2012
miR-1280	2Gy	1h	gamma	CD34+	+	Li, Ha 2012
miR-1281	2Gy	1h	gamma	CD34+	-	Li, Ha 2012
miR-1305	8Gy	1h	gamma	hFOB	+	Li, Ha 2012
miR-1826	8Gy	1h	gamma	hFOB	+	Li, Ha 2012
miR-1839-3p	4.3Gy	24h	^{177}Lu-octreotate	mouse renal cortical tissue	+	present study
miR-1902	13 Gy	24h	^{177}Lu-octreotate	mouse renal cortical tissue	-	present study
miR-1915	2Gy	1h	gamma	CD34+	-	Li, Ha 2012
miR-1976	2Gy	1h	gamma	CD34+	+	Li, Ha 2012
miR-2137	4.3Gy	24h	^{177}Lu-octreotate	mouse renal cortical tissue	-	present study
miR-2861	2Gy	1h	gamma	CD34+	-	Li, Ha 2012
miR-2861	1.3, 4.3Gy	24h	^{177}Lu-octreotate	mouse renal cortical tissue	+	present study
miR-3074	8Gy	1h	gamma	hFOB	-	Li, Ha 2012
miR-3076-3p	2 Gy	4h	proton	Mouse brain	-	Khan, Tariq 2013
miR-3077-5p	0.34, 1.3, 4.3, 13Gy	24h	^{177}Lu-octreotate	mouse renal cortical tissue	+	present study
miR-3084-3p	0.34, 4.3Gy	24h	^{177}Lu-octreotate	mouse renal cortical tissue	+	present study
miR-3090-5p	0.34, 1.3, 4.3, 13Gy	24h	^{177}Lu-octreotate	mouse renal cortical tissue	+	present study
miR-3096b-3p	13Gy	24h	^{177}Lu-octreotate	mouse renal cortical tissue	+	present study
miR-3102-5p	0.34, 4.3, 13Gy	24h	^{177}Lu-octreotate	mouse renal cortical tissue	+	present study
miR-3141	2Gy	1h	gamma	CD34+	-	Li, Ha 2012
miR-3185	8Gy	1h	gamma	hFOB	+	Li, Ha 2012
miR-3196	2Gy	1h	gamma	CD34+	-	Li, Ha 2012
miR-3470a	2 Gy	4h	proton	Mouse liver	+	Khan, Tariq 2013
miR-3470b	2 Gy	4h	proton	Mouse liver	+	Khan, Tariq 2013
miR-3473b	2 Gy	4h	proton	Mouse liver	+	Khan, Tariq 2013

miR-4267	8Gy	1h	gamma	hFOB	-	Li, Ha 2012
miR-4281	2Gy	1h	gamma	CD34+	-	Li, Ha 2012
miR-4298	8Gy	1h	gamma	hFOB	+	Li, Ha 2012
miR-5126b	8Gy	1h	gamma	hFOB	-	Li, Ha 2012
miR-5627-5p	4.3Gy	24h	^{177}Lu-octreotate	mouse renal cortical tissue	+	present study
miR-6239	13Gy	24h	^{177}Lu-octreotate	mouse renal cortical tissue	-	present study
miR-6240	4.3Gy	24h	^{177}Lu-octreotate	mouse renal cortical tissue	+	present study
miR-6244	0.34, 4.3, 13Gy	24h	^{177}Lu-octreotate	mouse renal cortical tissue	+	present study
miR-6402	1.3, 4.3, 13Gy	24h	^{177}Lu-octreotate	mouse renal cortical tissue	+	present study
miR-6538	13Gy	24h	^{177}Lu-octreotate	mouse renal cortical tissue	+	present study

Cha, H. J., K. M. Seong, S. Bae, J. H. Jung, C. S. Kim, K. H. Yang, Y. W. Jin and S. An (2009). "Identification of specific microRNAs responding to low and high dose gamma-irradiation in the human lymphoblast line IM9." Oncol Rep **22**(4): 863-868.

Cha, H. J., S. Shin, H. Yoo, E. M. Lee, S. Bae, K. H. Yang, S. J. Lee, I. C. Park, Y. W. Jin and S. An (2009). "Identification of ionizing radiation-responsive microRNAs in the IM9 human B lymphoblastic cell line." Int J Oncol **34**(6): 1661-1668.

Chen, G., W. Zhu, D. Shi, L. Lv, C. Zhang, P. Liu and W. Hu (2010). "MicroRNA-181a sensitizes human malignant glioma U87MG cells to radiation by targeting Bcl-2." Oncol Rep **23**(4): 997-1003.

Dickey, J. S., F. J. Zemp, O. A. Martin and O. Kovalchuk (2011). "The role of miRNA in the direct and indirect effects of ionizing radiation." Radiat Environ Biophys **50**(4): 491-499.

Girardi, C., C. De Pitta, S. Casara, G. Sales, G. Lanfranchi, L. Celotti and M. Mognato (2012). "Analysis of miRNA and mRNA expression profiles highlights alterations in ionizing radiation response of human lymphocytes under modeled microgravity." PLoS One **7**(2): e31293.

Hu, Z., Y. Tie, G. Lü, H. Fu, R. Xing, J. Zhu, Z. Sun and X. Zheng (2013). "Correlation of microRNAs responding to high dose γ-irradiation with predicted target mRNAs in HeLa cells using microarray analyses." Chinese Science Bulletin **58**(36): 4622-4629.

Ilnytskyy, Y., F. J. Zemp, I. Koturbash and O. Kovalchuk (2008). "Altered microRNA expression patterns in irradiated hematopoietic tissues suggest a sex-specific protective mechanism." Biochem Biophys Res Commun **377**(1): 41-45.

Joly-Tonetti, N., J. Vinuelas, O. Gandrillon and J. Lamartine (2013). "Differential miRNA expression profiles in proliferating or differentiated keratinocytes in response to gamma irradiation." BMC Genomics **14**: 184.

Josson, S., S. Y. Sung, K. Lao, L. W. Chung and P. A. Johnstone (2008). "Radiation modulation of microRNA in prostate cancer cell lines." Prostate **68**(15): 1599-1606.

Khan, S. Y., M. A. Tariq, J. P. Perrott, C. D. Brumbaugh, H. J. Kim, M. I. Shabbir, G. T. Ramesh and N. Pourmand (2013). "Distinctive microRNA expression signatures in proton-irradiated mice." Mol Cell Biochem **382**(1-2): 225-235.

Koturbash, I., F. Zemp, B. Kolb and O. Kovalchuk (2011). "Sex-specific radiation-induced microRNAome responses in the hippocampus, cerebellum and frontal cortex in a mouse model." Mutat Res **722**(2): 114-118.

Leung, C. M., S. C. Li, T. W. Chen, M. R. Ho, L. Y. Hu, W. S. Liu, T. T. Wu, P. C. Hsu, H. T. Chang and K. W. Tsai (2014). "Comprehensive microRNA profiling of prostate cancer cells after ionizing radiation treatment." Oncol Rep **31**(3): 1067-1078.

Li, B., X. B. Shi, D. Nori, C. K. Chao, A. M. Chen, R. Valicenti and V. White Rde (2011). "Down-regulation of microRNA 106b is involved in p21-mediated cell cycle arrest in response to radiation in prostate cancer cells." Prostate **71**(6): 567-574.

Li, X. H., C. T. Ha, D. Fu and M. Xiao (2012). "Micro-RNA30c negatively regulates REDD1 expression in human hematopoietic and osteoblast cells after gamma-irradiation." PLoS One **7**(11): e48700.

Maes, O. C., J. An, H. Sarojini, H. Wu and E. Wang (2008). "Changes in MicroRNA expression patterns in human fibroblasts after low-LET radiation." J Cell Biochem **105**(3): 824-834.

Nikiforova, M. N., M. Gandhi, L. Kelly and Y. E. Nikiforov (2011). "MicroRNA dysregulation in human thyroid cells following exposure to ionizing radiation." Thyroid **21**(3): 261-266.

Shin, S., H. J. Cha, E. M. Lee, S. J. Lee, S. K. Seo, H. O. Jin, I. C. Park, Y. W. Jin and S. An (2009). "Alteration of miRNA profiles by ionizing radiation in A549 human non-small cell lung cancer cells." Int J Oncol **35**(1): 81-86.

Simone, N. L., B. P. Soule, D. Ly, A. D. Saleh, J. E. Savage, W. Degraff, J. Cook, C. C. Harris, D. Gius and J. B. Mitchell (2009). "Ionizing radiation-induced oxidative stress alters miRNA expression." PLoS One **4**(7): e6377.

Templin, T., S. A. Amundson, D. J. Brenner and L. B. Smilenov (2011). "Whole mouse blood microRNA as biomarkers for exposure to gamma-rays and (56)Fe ion." Int J Radiat Biol **87**(7): 653-662.

Templin, T., S. Paul, S. A. Amundson, E. F. Young, C. A. Barker, S. L. Wolden and L. B. Smilenov (2011). "Radiation-induced micro-RNA expression changes in peripheral blood cells of radiotherapy patients." Int J Radiat Oncol Biol Phys **80**(2): 549-557.

Templin, T., E. F. Young and L. B. Smilenov (2012). "Proton radiation-induced miRNA signatures in mouse blood: characterization and comparison with 56Fe-ion and gamma radiation." Int J Radiat Biol **88**(7): 531-539.

Wagner-Ecker, M., C. Schwager, U. Wirkner, A. Abdollahi and P. E. Huber (2010). "MicroRNA expression after ionizing radiation in human endothelial cells." Radiat Oncol **5**: 25.

Weidhaas, J. B., I. Babar, S. M. Nallur, P. Trang, S. Roush, M. Boehm, E. Gillespie and F. J. Slack (2007). "MicroRNAs as potential agents to alter resistance to cytotoxic anticancer therapy." Cancer Res **67**(23): 11111-11116.
